# Spectral Responses of Urban and Peri-Urban Woody Canopy to Recurrent Drought: A Matched Three-City Pilot

**DOI:** 10.3390/plants15213401

**Published:** 2026-11-02

**Authors:** Sinan Kordon, Erdi Ekren, Emre Babur

**Affiliations:** 1 Department of Landscape Architecture, Faculty of Forestry, Kahramanmaraş Sütçü İmam University, Avşar Campus, 46050 Kahramanmaraş, Türkiye; eekren@ksu.edu.tr; 2 Department of Forest Engineering, Faculty of Forestry, Kahramanmaraş Sütçü İmam University, Avşar Campus, 46050 Kahramanmaraş, Türkiye

**Keywords:** recurrent drought, urban forests, peri-urban vegetation, woody canopy, Harmonized Landsat–Sentinel-2, spectral anomalies, matched comparisons, SPEI

## Abstract

Urban conditions may increase woody-vegetation drought stress, although management and canopy composition can modify urban–peri-urban differences. We compared spectral anomalies of persistent woody canopy in Berlin, Madrid, and Vienna using Harmonized Landsat–Sentinel-2 observations from 2013 to 2024. Seventy urban–peri-urban pairs were matched on elevation, long-term temperature, precipitation, and retained woody area. Of 26 predefined candidate recurrent-drought events, 12 met the 80% observation-support criterion. Outcomes were event medians of within-pair urban-minus-peri-urban anomalies in the two-band enhanced vegetation index (EVI2) and normalized difference moisture index (NDMI). Each index produced seven positive and five negative contrasts; medians were 0.00047 for EVI2 and 0.00065 for NDMI. Event-level models adjusted for city and current drought severity examined antecedent severity and inter-event interval. The standardized predictor coefficients ranged from −0.00225 to 0.00252 in terms of index units. All four heteroskedasticity-consistent 95% confidence intervals included zero. The same pattern emerged in pair-within-event resampling, spatial buffering, and models based on growing-season severity, with every interval crossing zero. As the primary regressions contained 12 events and five fitted terms, there were only seven residual degrees of freedom, resulting in imprecise estimates and low statistical power. Climate antecedents were identifiable for all 26 candidates, and analytical inclusion was determined by vegetation observation support. Across the retained cases, we found neither a consistent urban spectral disadvantage nor a clear relationship between the contrast and drought history. The matched design is reproducible, but the evidence it provides comes from a small sample selected based on observation availability and from dense canopies that persisted throughout the study period. Accordingly, these estimates should not be generalized to whole urban forests or interpreted as measures of physiological response, mortality, or canopy loss.

## 1. Introduction

Ecological responses can outlast the meteorological drought itself. Factors such as incomplete canopy recovery, hydraulic injury, altered resource allocation, changes in community composition, and altered soil processes may influence the response to subsequent events [1,2,3]. While a prior drought can intensify subsequent damage, acclimation, selective survival, and compositional changes may also mitigate it [2,3]. Therefore, a recurrent drought has no predetermined response direction.

These distinctions also depend on terminology and measurement. Resistance, recovery, legacy effects, and ecological memory describe related, but non-equivalent, aspects of drought response [4,5,6]. An association with antecedent climate alone is not evidence of physiological memory.

Earth observation indices provide an additional layer of interpretation. Greenness and moisture-sensitive metrics, for example, describe different canopy properties, and the choice of resilience metric can alter the resulting spatial pattern [7].

While urban woody vegetation experiences the same regional hydroclimatic anomalies as peri-urban vegetation, it also grows in conditions of urban heat, restricted rooting volumes, altered soils, fragmentation and uneven management. These conditions may heighten drought sensitivity. However, in other settings, factors such as irrigation, species selection, local microclimate or maintenance may counteract or even reverse the expected urban–peri-urban contrast. Accordingly, tree-ring and remote-sensing studies report substantial variation among sites, species, and cities [8,9,10]. Recent multi-city studies have quantified urban vegetation resistance and recovery across broad domains [11,12], but event counts alone cannot describe the sequence of events immediately preceding a current drought.

Broad-domain comparisons do not by themselves distinguish measurement density from exposure replication. Thousands of pixels can characterize canopy condition within an event, yet those pixels share a regional drought history. A matched design can reduce regional exposure differences while treating each city-event, rather than each pixel or matched pair, as the unit of exposure analysis. City-events can nevertheless share temporal and regional dependence. We therefore combined outcome-blind urban–peri-urban matching with exploratory event-level models to examine associations between drought-sequence attributes and within-event spectral contrasts.

A matched urban–peri-urban design can reduce regional climate differences while preserving the local-context contrast, but it cannot eliminate unmeasured differences in species, soil, disturbance, or management. We therefore treated Berlin, Madrid, and Vienna as contrasting comparative cases rather than a representative European sample.

We asked: (1) how urban and peri-urban persistent woody-canopy anomalies varied among recurrent-drought events and indices; (2) whether urban-minus-peri-urban anomalies were consistently associated with antecedent drought severity or inter-event interval after adjustment for current-event severity and city; and (3) whether those associations remained stable under alternative temporal aggregation, event spacing, measurement resampling, spatial buffering, and growing-season-aligned severity. We treated the three cities as comparative cases and used additional robustness and design diagnostics to define the limits of inference.

## 2. Results

### 2.1. Analytical Sample and Matching

**Figure 1:**
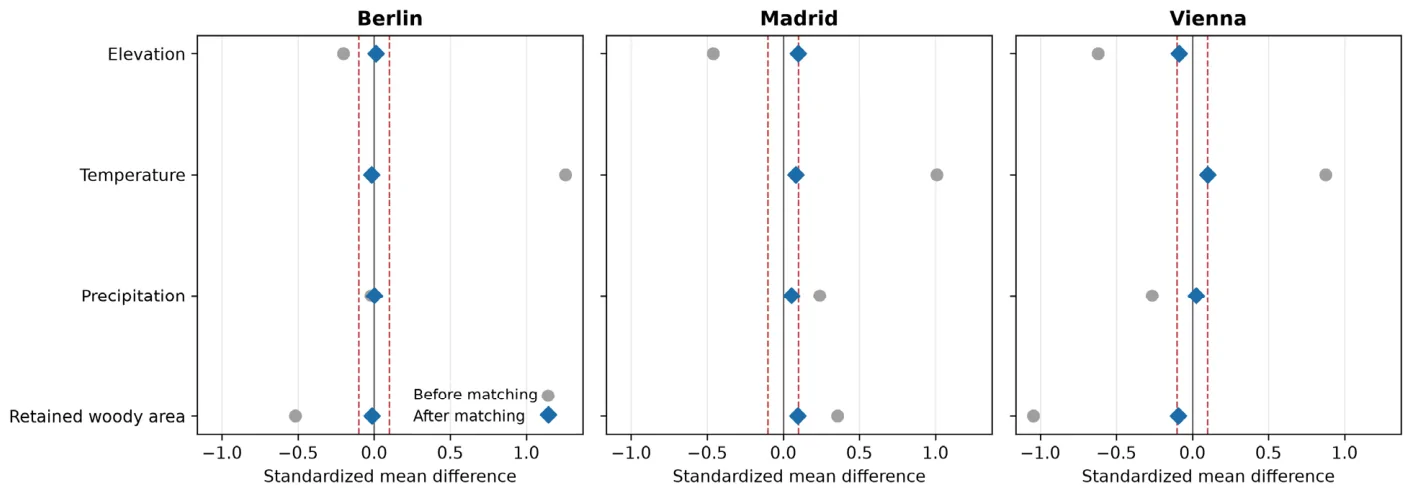
Covariate balance before and after matching in the three analytical cities. Dashed lines mark the defined absolute standardized mean difference threshold of 0.10. Standardization used the fixed candidate-sample standard deviation.

All 26 candidate current events had an immediately preceding qualifying event in the complete 1961–2024 climate record. Fourteen current events failed the predefined 80% observation-support criterion, leaving 12 analytical events: two in Berlin, five in Madrid, and five in Vienna. Five events had antecedents predating the HLS response period, but all five independently failed the vegetation-support criterion; therefore, use of the full climate record did not alter the 12-event analytical sample. The three cities contributed 140 matched units, 70 matched pairs, 16,721 persistent woody pixels, and 1399.96 ha of exact clipped woody area (Table 1). Post-match maximum absolute standardized mean differences for the four matching covariates were 0.0175 in Berlin, 0.0980 in Madrid, and 0.0995 in Vienna (Figure 1).

The retained events spanned current full-run severities ranging from 0.344 to 9.318 and antecedent full-run severities ranging from 0.601 to 9.318. The inter-event intervals ranged from 2 to 30 months (Table 2). Growing-season-aligned current severities ranged from 0.344 to 6.871 and aligned antecedent severities from 0.601 to 6.871. The design-fixed denominator comprised 260 pair-event records (60 Berlin, 50 Madrid, and 150 Vienna). A prefit raw-support screen made one Madrid 2018–12 pair ineligible for retrieval, leaving 259 retrievable records; consequently, row-based derived files that begin after this screen sum to 259 rather than 260. Final reference and outcome support retained 253 records. Both Berlin events began with 30 design-fixed pairs and passed the early raw month-support screen for all 30, but only 27 per event met the later reference/outcome support requirements. Madrid retained 9 of 10 pairs for 2018–12 and 10 of 10 for the other four events (49/50 overall), while Vienna retained all 30 pairs in each event. No replacement pairs were introduced, and the unavailable Madrid record remains in the fixed denominator.

### 2.2. Urban and Peri-Urban Anomalies

**Figure 2:**
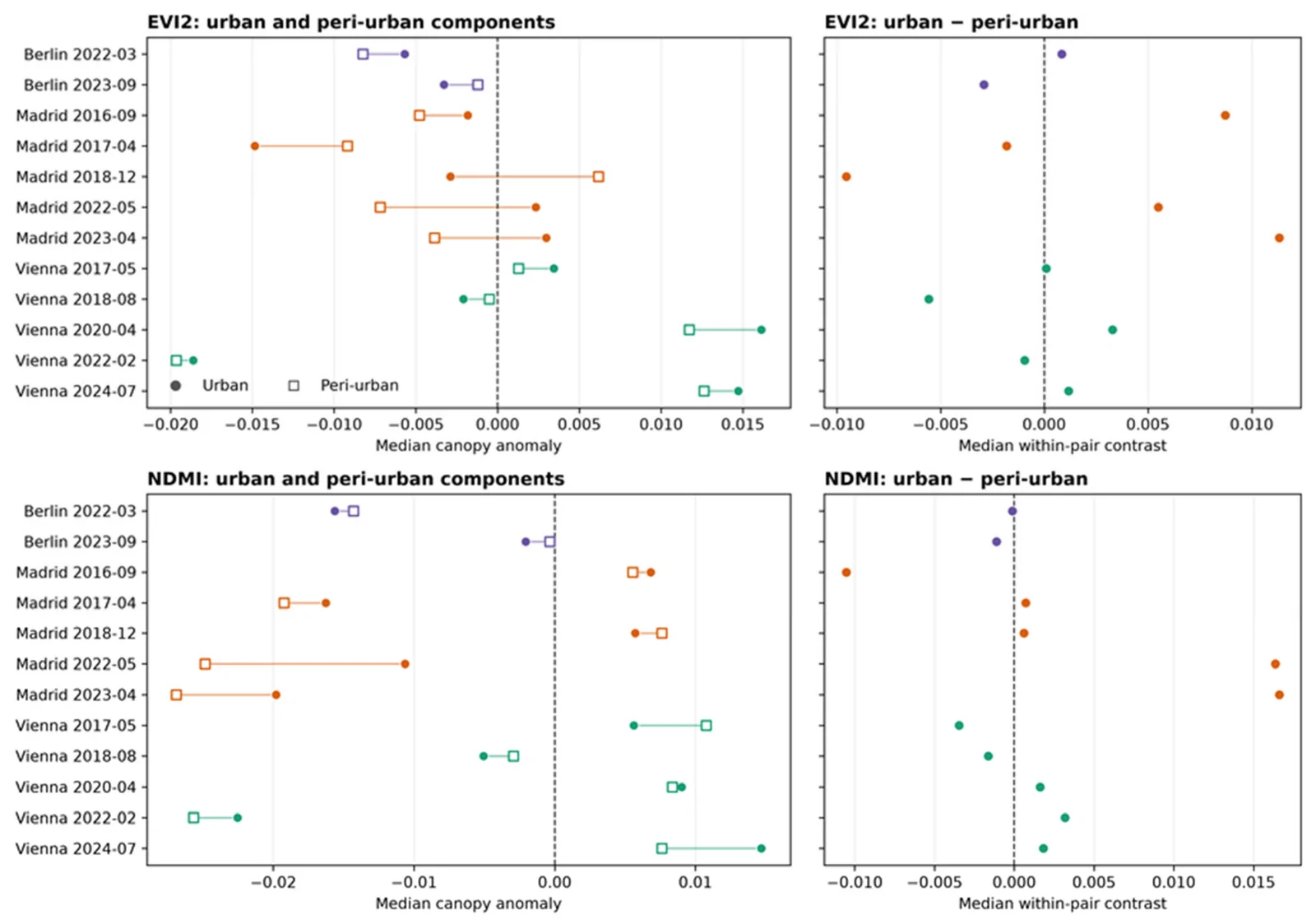
Event-level equal-month EVI2 and NDMI anomalies. Colours identify the analytical cities: purple denotes Berlin, orange denotes Madrid, and green denotes Vienna. Filled circles denote urban components, open squares denote peri-urban components, and the right panels show median within-pair urban-minus-peri-urban contrasts. The zero line indicates equal anomalies rather than an absence of drought response in both components.

Under equal-month aggregation, EVI2 showed seven positive and five negative urban-minus-peri-urban contrasts (median 0.00047; range −0.00955 to 0.01133). The Normalised Difference Moisture Index (NDMI) showed the same count by sign, with a median value of 0.00065 and values ranging from −0.01054 to 0.01660. Urban and peri-urban component anomalies were often similar, although their difference changed in size and direction across events (Figure 2). These are within-design spectral contrasts, not causal urban effects. Positive values simply indicate that the urban anomaly exceeded the peri-urban anomaly; neither component has to be positive. Similarly, a contrast near zero may indicate similar declines in both components.

### 2.3. Drought-Sequence Associations

All four primary estimates were small relative to their uncertainty intervals (Table 3). For antecedent severity, a one-standard deviation (SD) increase corresponded to −0.00225 in the EVI2 contrast and 0.00180 in the normalized difference moisture index (NDMI) contrast. A one-standard deviation (SD) increase in log interval corresponded to 0.00071 for EVI2 and 0.00252 for NDMI. The value of zero lay within every HC3 95% interval. The NDMI antecedent estimate kept the same sign in all deletion fits, whereas each of the other three changed sign in at least one fit. These coefficients refer to the urban-minus-peri-urban contrast and not to either component alone. The intervals also do not demonstrate equivalence between the two settings.

### 2.4. Sensitivity to Temporal Definitions and Event Spacing

**Figure 3:**
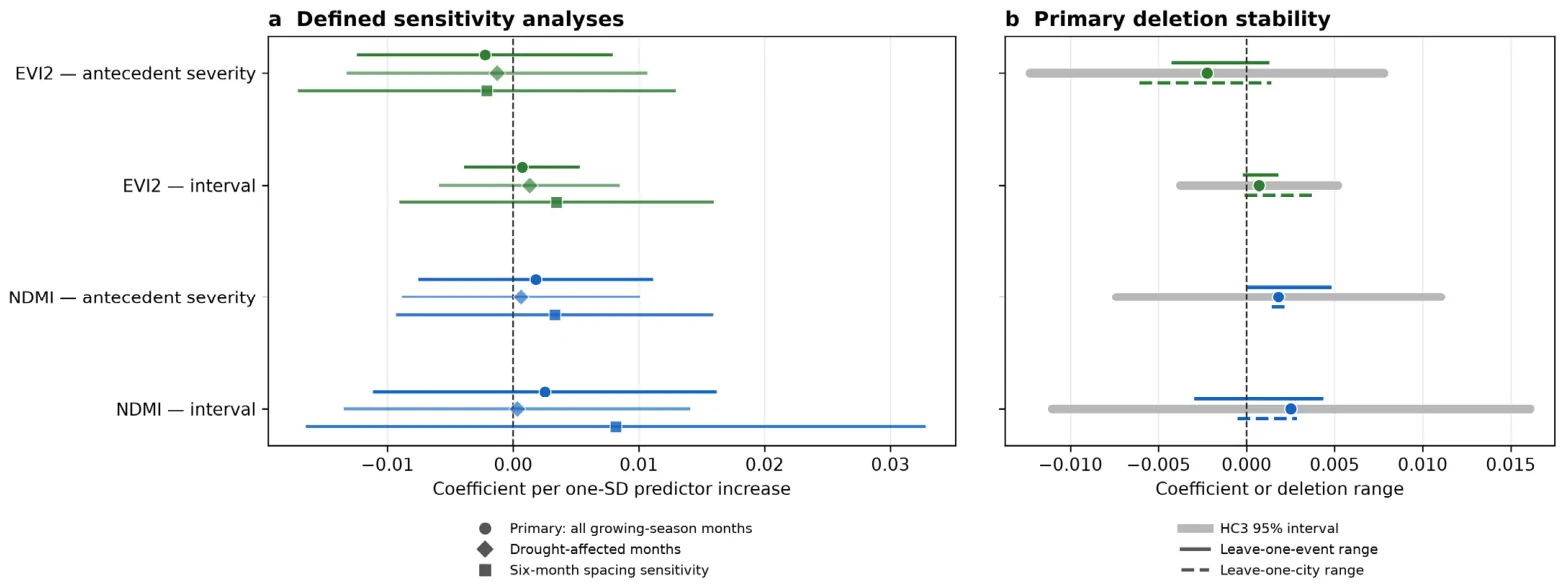
(**a**) Focal coefficients and HC3 95% intervals from the primary, affected-month, and six-month-spacing analyses; circles, diamonds, and squares denote the respective point estimates. (**b**) Primary point estimates, shown as circles, and their HC3 95% intervals, together with the ranges obtained after removing each event or each city in turn. Colours distinguish the vegetation indices, while solid and dashed lines denote the leave-one-event and leave-one-city ranges, respectively.

The analyses of equal valid days, acquisition weights and affected months gave the same four directions of coefficient as the full sample, but every HC3 interval crossed zero. Requiring at least six months between successive events excluded Madrid 2017–04 and Madrid 2023–04. The resulting ten-event models had five residual degrees of freedom and yielded four intervals containing zero (see Table 4 and Figure 3). With two fewer events, the precision and power deteriorated further, leaving the sign and size of the focal associations unresolved.

### 2.5. Extended Robustness and Sensitivity

**Figure 4:**
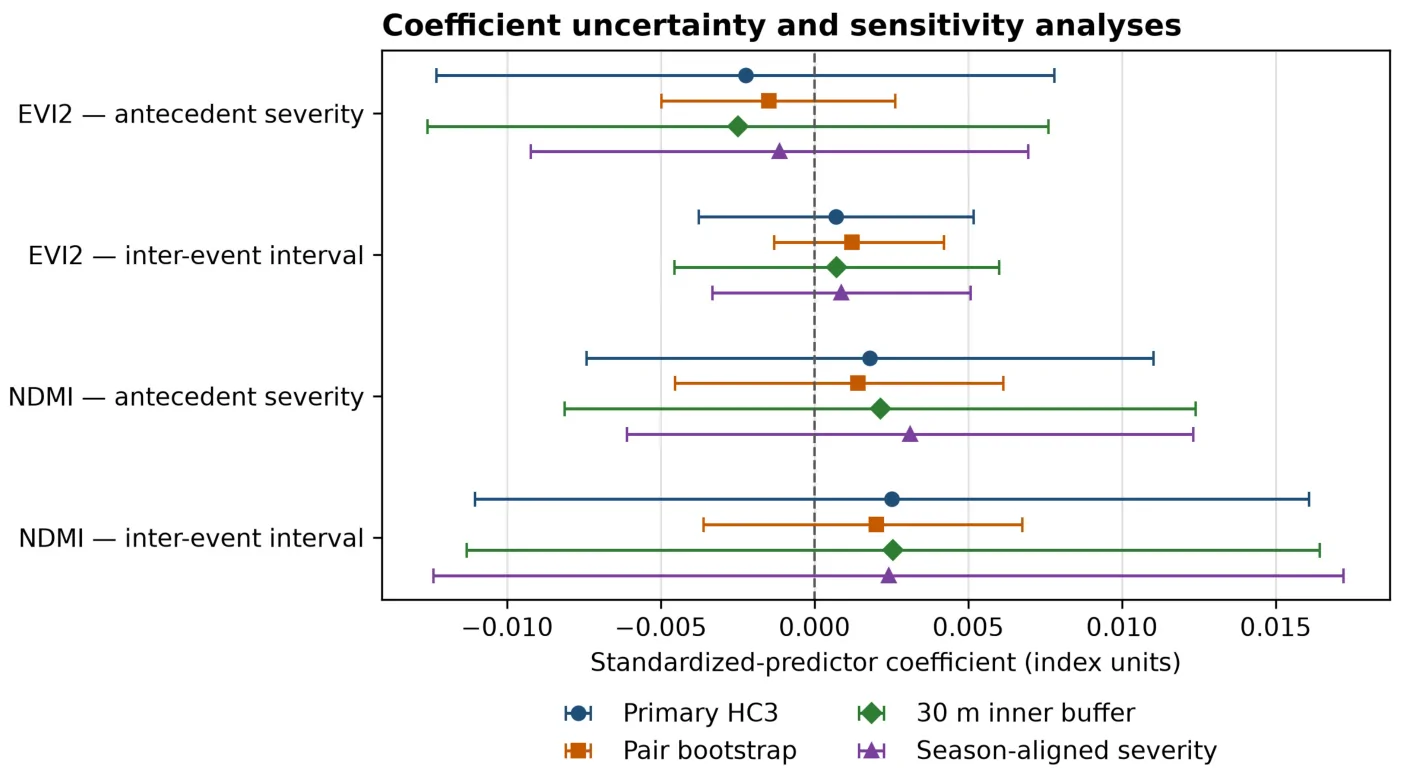
Primary HC3 intervals, intervals from 10,000 pair-within-event bootstrap replicates, 30 m inner-buffer HC3 intervals, and HC3 intervals based on growing-season-aligned severity; all are conditional on the same 12 events.

The pair-within-event bootstrap retained uncertainty around zero for every focal coefficient: percentile intervals were −0.00497 to 0.00263 for EVI2 antecedent severity, −0.00132 to 0.00421 for EVI2 interval, −0.00454 to 0.00615 for NDMI antecedent severity, and −0.00360 to 0.00675 for NDMI interval (Table 5; Figure 4). These intervals quantify pair aggregation uncertainty conditional on the retained events.

The geometry-stage 30 m inner-buffer audits evaluated both unit members of all 260 design-fixed pair-event records (520 unit-event cases) and retained 511. Outcome reconstruction then rejoined the frozen 259 retrievable pair-event records; the prefit-ineligible Madrid 2018-12 pair remained unavailable. Fixed reference/pair support and the unchanged 0.80 event gate retained all 12 events. The four focal estimates were −0.00249, 0.00072, 0.00214, and 0.00256 in Table 5 order; none changed sign, and every HC3 interval included zero. Growing-season-aligned severity also preserved all four primary directions. Its focal estimates were −0.00113 for EVI2 antecedent severity, 0.00088 for EVI2 interval, 0.00311 for NDMI antecedent severity, and 0.00241 for NDMI interval; all four HC3 intervals included zero.

No event exceeded the 2*p*/*n* leverage threshold. Depending on the primary model, one to three events exceeded the 4/*n* Cook’s-distance threshold, and deletion analyses showed that individual events remained consequential. No flagged event was removed.

### 2.6. Design Limits and Audit Results

Median distances between matched-pair centroids were 3.28 km in Berlin, 10.73 km in Madrid, and 6.54 km in Vienna; the ranges extended to 47.19 km, 20.23 km, and 30.31 km, respectively. All four of the matching covariates satisfied the predefined post-match balance rule. As intended, the built-fraction descriptor remained higher in urban units, consistent with the GHS-SMOD settlement contrast; it was not used to rematch units after outcomes were seen.

The two-harmonic model without a trend was chosen for all the events in Berlin and most of those in Madrid and Vienna. Across city-index groups, median selected held-out RMSE ranged from 0.0399 to 0.0523. Those values describe pixel-day model selection and should not be compared directly with event-median urban-minus-peri-urban contrasts. To examine the use of current-event holdouts during complexity selection, we recalculated every outcome with a one-harmonic no-trend reference fixed in advance. All 12 events remained, supported by the same 259 retrievable pair-event records. The equal-month focal estimates were 0.00065 (95% HC3 interval −0.00826 to 0.00956) for EVI2 antecedent severity, 0.00319 (−0.00507 to 0.01145) for EVI2 interval, 0.00391 (−0.00515 to 0.01298) for NDMI antecedent severity, and 0.00407 (−0.00557 to 0.01371) for NDMI interval. Every interval included zero, although the EVI2 antecedent estimate changed sign relative to the primary model. Thus, the overall absence of directional evidence remained unchanged, whereas the sign of an individual coefficient depended on the form of the reference. It was not possible to fit a completely drought-free reference while retaining the predefined minimum of eight training years: only two candidate years were available in Berlin, and four in each of Madrid and Vienna.

The same caution applied to sensor composition. Across the 12 retained events, the median pair-level L30 share in inventories for the current affected months ranged from 0.1415 to 0.4639. After standardized event-level L30 share was added to each primary model, six residual degrees of freedom remained. The adjusted focal estimates were −0.00421 for EVI2 antecedent severity, −0.00026 for EVI2 interval, 0.00056 for NDMI antecedent severity and 0.00177 for NDMI interval. Every HC3 interval crossed zero. The EVI2 interval estimate, already close to zero, became negative. In the raw-support screens, only two of the 12 L30-only events met the 80% pair-support threshold, compared to 11 of the 12 S30-only events and all 12 events for the combined product. The bounded same-day comparison contained one jointly valid date and nine jointly valid pixels, all of which were from a single peri-urban unit. The mean L30 minus S30 differences were 0.00913 for EVI2 and 0.00590 for NDMI. This limited comparison cannot establish general equivalence between sensors, and sensor-specific reruns of the outcome were not interpreted as comparable 12-event analyses.

**Figure 5:**
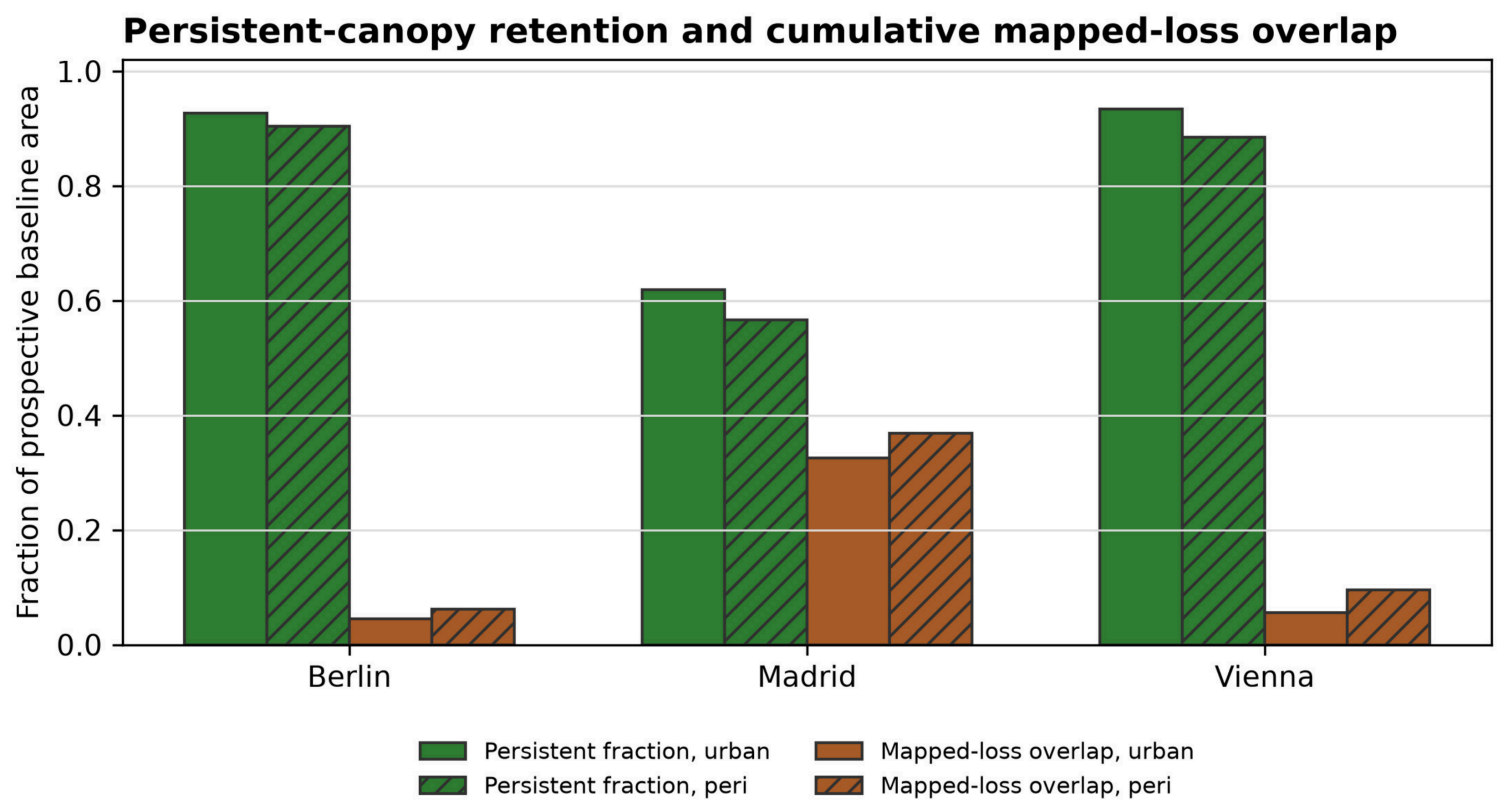
Persistent-canopy retention and overlap with cumulative mapped loss, expressed as fractions of prospective baseline woody area for each city and stratum. The mapped loss should not be interpreted as drought-attributable.

The persistent area represented 0.567–0.934 of the prospective baseline woody area across the city stratum groups (Figure 5), while the cumulative mapped loss overlap represented 0.045–0.368. These quantities characterize survivor conditioning and do not identify canopy loss caused by drought. The specified drought-run definition yielded different climate-only event inventories under SPEI-3, SPEI-6, and SPEI-12. Vegetation outcomes were not recomputed for these alternative accumulation windows.

Among the 12 events, antecedent severity was only weakly correlated with log interval, whereas current severity was strongly correlated with event duration. Although six events met the current-plus-post support criterion, their first post-event observations did not provide enough information to estimate recovery duration or a causal post-drought effect. The supplement provides detailed audits of reference fit, residuals, equivalence, power, influence and event definition.

## 3. Discussion

Across matched cases, each index had seven positive and five negative urban-minus-peri-urban contrasts, and both medians were near zero. We therefore found no consistent spectral disadvantage for persistent urban canopy in the retained cases from Berlin, Madrid, and Vienna. This pattern does not show that urban and peri-urban drought sensitivity is equal. The five-parameter models were fitted to only 12 city-events, leaving seven residual degrees of freedom. Consequently, precision and power were low: plausible effects extend in both directions, and similar declines in the two components may cancel in the contrast.

Pair-level resampling, alternative temporal aggregation, a six-month spacing rule, a 30 m inward buffer, and growing-season-aligned severity all yielded focal intervals spanning zero. The spatial and climate-alignment sensitivities preserved the four primary coefficient directions. While this provides limited consistency across specified analytical choices, sign preservation does not establish a supported association. The inward buffer only addresses unit boundaries, not all mixed-pixel contamination. Furthermore, seasonal alignment does not resolve exposure uncertainty. Neither analysis increase exposure replication.

The fixed-reference and sensor-composition checks add uncertainty rather than evidence for a common direction. All relevant HC3 intervals crossed zero. Moreover, the EVI2 antecedent estimate changed sign under the fixed one-harmonic reference, and the near-zero EVI2 interval estimate changed sign after sensor adjustment. Support from the two sensors was uneven, while the same-day comparison contained too little information to assess equivalence. The most defensible result is therefore the lack of directional evidence; the signs of individual coefficients are less robust.

All 26 candidate events had identifiable antecedents in the full climate record from 1961 to 2024. Five antecedents predated the HLS response period; however, all five corresponding current events failed the predefined vegetation-support criterion independently. Therefore, antecedent eligibility was determined from climate history, while vegetation support determined the 12-event analytical sample. However, observation-based selection can still affect representativeness; events with adequate clear-sky and reference support may differ from excluded events. Therefore, the findings apply to the retained cases rather than to all 26 candidates.

Drought severity and event spacing antecedents were not consistently associated with the observed contrasts. The EVI2 antecedent estimate was negative, and the NDMI estimate was positive under both full-run and growing-season-aligned severity; however, their intervals included zero. These exploratory associations do not measure physiological memory, and the current sample is too small to distinguish weak associations from insufficient precision.

The study contains many measurements, but few independent exposure histories. Canopy condition was measured from thousands of pixels and summarized in 253 pair-event records, yet these reduce to 12 city events in three cities. Therefore, treating pixels, pairs, or bootstrap draws as separate drought histories would exaggerate replication. Event-level models prevent pixel-level pseudoreplication, and the conditional bootstrap tests sensitivity to pair aggregation. However, neither method removes dependence between repeated events in one city or between simultaneous regional droughts.

Although matching achieved a balance in terms of elevation, long-term temperature and precipitation, and retained woody area, it did not control every difference between locations. The built fraction remained deliberately higher in urban units because it reflected the intended settlement contrast. Pair distance varied across each functional urban area and no data were available on species, irrigation, management, soil conditions or fine-scale heat. In urban pixels, factors such as irrigation, species choice, pruning, modified rooting space, compacted soils, and artificial water tables may weaken, hide, or reverse contrasts associated with meteorological drought. Therefore, the estimates are adjusted spatial comparisons rather than causal effects of urbanization.

EVI2 and NDMI describe different canopy properties and do not necessarily move together. EVI2 is influenced by greenness and canopy structure, while NDMI combines NIR and SWIR1 reflectance and is sensitive to water-related spectral properties [13,14,15]. Both are affected by canopy composition, structure, and subpixel mixtures. Water-use strategies range from more isohydric to more anisohydric behaviour and differ among species, potentially shifting with environmental conditions [16]. Differences in species composition or management between urban and peri-urban areas could therefore reduce or reverse the contrast, even under the same regional meteorological drought. Neither index measures hydraulic function, stomatal behaviour, mortality, irrigation, soil moisture, or ecological memory directly. Differences between indices cannot identify a mechanism; instead, they justify retaining both prespecified outcomes rather than choosing the one with a preferred sign.

The selection of the surviving canopy defines the population represented here. The requirement of high historical tree cover, overlap with the 2020 tree classification and the absence of mapped loss through to 2024 improved temporal comparability, but excluded canopies that did not persist. Retention rates varied between cities, being lowest in Madrid. Accordingly, the reported anomalies pertain only to surviving, high-density woody patches. They do not describe the health of the entire urban forest, tree mortality, canopy clearing or patches lost before the outcome period. The excluded trajectories may have differed systematically.

Interpretation also depends on how the reference and climate exposure were defined. Current event years were excluded from their reference fits, but other drought years could remain in the training series. Removing every qualifying drought year resulted in too few years remaining for fitting the reference. Alternative SPEI accumulation windows produced materially different event inventories. Rerunning the complete outcomes for these windows would require the construction of new references, supports and footprints, and this could not be approximated by relabelling existing outcomes.

These results have a narrow practical implication for assessment design. In the retained cases, an urban location did not reliably predict a lower spectral anomaly. This evidence is insufficient to alter irrigation practices, select species, or rank city drought resilience. Explanatory studies would require species and management inventories, high-resolution soil moisture data, localized land surface temperature data, rooting space and soil information, and data on the timing of canopy loss. Confirmation would require more cities and city-event histories, models representing dependence, and a direct comparison of supported and excluded events under identical outcome-blind rules. If future work relaxes spatial or cloud support criteria, this should be specified before outcomes are inspected and reported as a sensitivity analysis.

The main limitations are the three observation-screened cities, the HLS response period from 2013 to 2024, the regional SPEI at 0.5°, changes in the availability of L30/S30, the absence of measurements of management, species composition, fine-scale heat, soil compaction, irrigation or artificial water tables, the selection of surviving canopies and the seven residual degrees of freedom in each primary model. Additionally, outcomes may be correlated due to the reuse of matched units and parts of the reference record across events. SPEI describes meteorological drought rather than root-zone water, and its regional grid cannot resolve urban hydrology or microclimate. City fixed effects account for average differences among cities, but they neither remove these dependencies nor create city-level replication. Therefore, the evidence should be interpreted as an exploratory comparison rather than as a European mean, an assessment of total urban forest health, or a demonstration of equivalent drought responses.

## 4. Materials and Methods

### 4.1. Study Design and Settlement Classes

**Figure 6:**
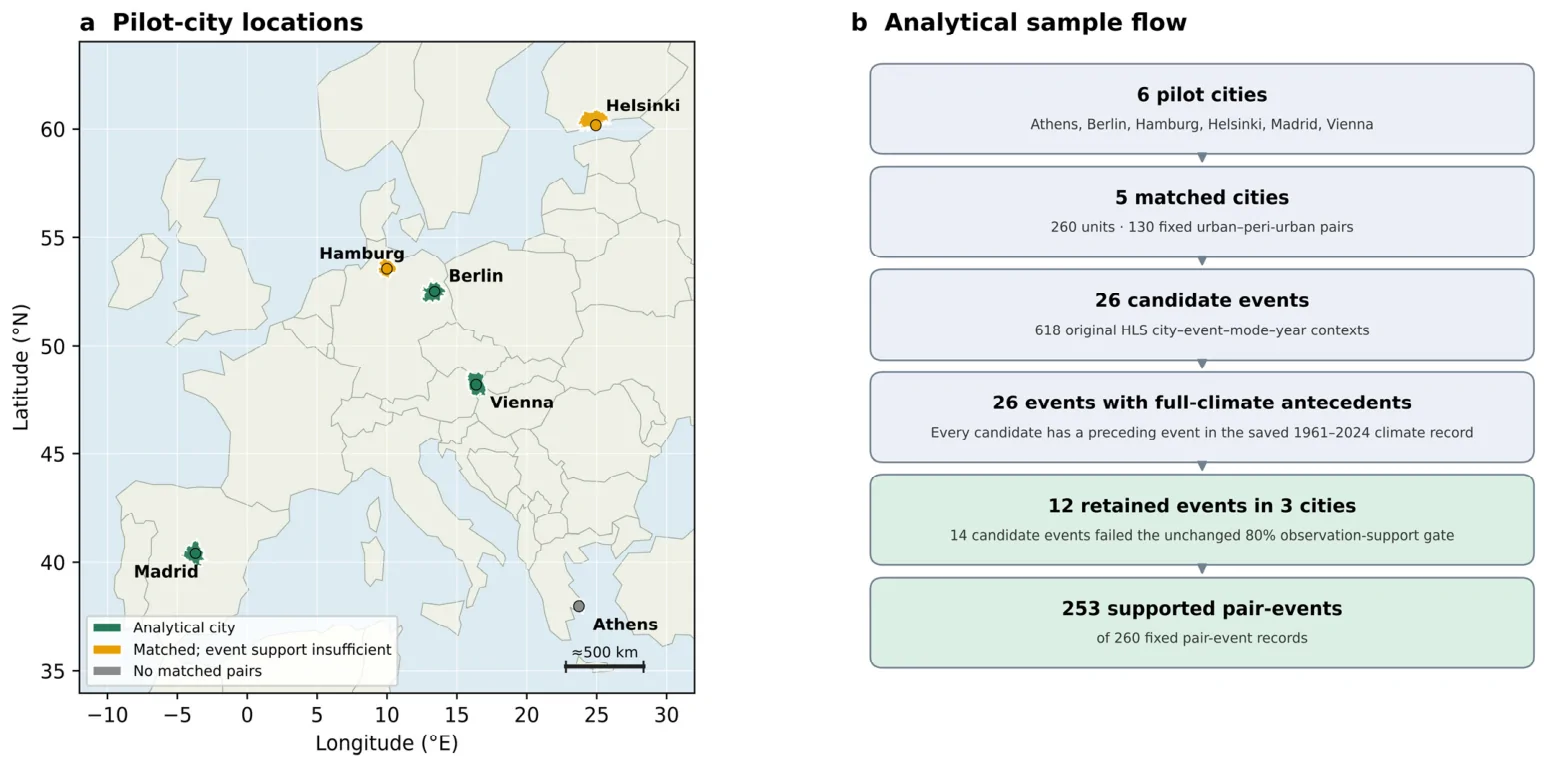
Pilot-city locations (**a**) and analytical sample flow (**b**). The map includes all six cities screened before analysis; retained events came from Berlin, Madrid, and Vienna. In panel (**a**), the basemap is the public-domain Natural Earth 1:110 m Admin 0 countries layer, and coloured outlines show the official Functional Urban Areas (WGS84; EPSG:4326). The scale bar is approximate. In panel (**b**), blue boxes represent the successive screening and eligibility stages, whereas green boxes identify the retained analytical sample and supported pair-event records. The audit trail retains every event and unsupported pair-event record. The audit trail retains every event and unsupported pair-event record.

Persistent settlement context was defined from the 1990 and 2020 GHS-SMOD epochs [17]. Urban units were class 23 (dense urban cluster) or class 30 (urban centre) at both dates; peri-urban units were class 21 (suburban or peri-urban grid cell) at both dates. Study domains used the Eurostat GISCO Urban Audit 2021 Functional Urban Areas, 1:100,000 generalized regions in WGS84 (EPSG:4326; https://ec.europa.eu/eurostat/web/gisco/geodata/statistical-units/cities-functional-urban-areas; downloaded and accessed on 16 September 2026). The configured city coordinates (longitude, latitude) were Athens (23.7275, 37.9838), Berlin (13.4050, 52.5200), Hamburg (9.9937, 53.5511), Helsinki (24.9384, 60.1699), Madrid (−3.7038, 40.4168), and Vienna (16.3738, 48.2082). The six-city screen was recorded on 16 September 2026 to span northern, central, and Mediterranean Europe and contrasting aridity before feasibility outcomes were known. Athens produced no feasible matched pairs. The predefined combined-HLS candidate set contained 26 events in the other five cities. Berlin, Madrid, and Vienna supplied the 12 events that met the observation-support criterion (Figure 6). The design is a matched comparative pilot, not a representative European sample.

### 4.2. Matching

Within each functional urban area, candidate urban and peri-urban units were defined as circles with a radius of 1 km and matched using mixed-integer linear programming. A match was admissible only when the units differed by no more than 100 m in elevation and 1.5 degrees Celsius in 1981–2010 mean temperature, when their 1981–2010 annual precipitation ratio did not exceed 1.2, and when the ratio of retained woody area did not exceed four. TerraClimate supplied the climate covariates [18], and elevation was taken from the mean band of USGS GMTED2010 (USGS/GMTED2010_FULL). Each admissible edge was assigned a cost equal to the sum of the squared covariate differences divided by the fixed variances of the candidate sample. To ensure computational tractability, the eligible edge set was restricted to the union of the 25 lowest-cost compatible alternatives from each side. The “25 nearest” solution was based on standardized covariate cost rather than geographic distance, meaning that it was only optimal within this restricted edge pool. The solver was scipy.optimize.milp under Python 3.13.14 and SciPy 1.18.1, with a 10 s limit for each requested pair count, relative MIP gap 0.01, one thread, and a balance-constraint numerical tolerance of 1 × 10^−6^. It sought the largest feasible sample from 30 pairs downward and required every post-match absolute standardized mean difference for the four matching covariates to be below 0.10. No pairs were replaced after the denominator was defined.

The 2020 built fraction was an urbanization descriptor used to characterize the intended settlement contrast; it was not a fifth balance target. Balancing it would have removed part of the exposure distinction defined by GHS-SMOD. Geographic distance was also not optimized, but pair-centroid distances were examined after matching. These choices reduce measured regional differences without making urban and peri-urban units exchangeable causal counterfactuals.

### 4.3. Persistent Woody-Canopy Cohort

Satellite processing used a fixed 30 m EPSG:3035 grid. Hansen 2000 tree cover was mean-aggregated to the analysis grid, and the ESA WorldCover 2020 tree class was converted to a fractional overlap at 30 m. Persistent woody pixels required Hansen tree cover of at least 70%, WorldCover tree fraction of at least 0.70, and Hansen lossyear not in 1–24, thereby excluding mapped loss during 2001–2024 while not using the product’s 2025 loss code [19,20]. The Hansen asset was UMD/hansen/global_forest_change_2025_v1_13. Eligible 1 km units required at least 1.8 ha of retained woody canopy. Earth Engine pixelArea intersected with the exact source geometry supplied within-unit area weights; these weights, rather than whole-pixel counts, were used in area summaries and outcomes. This survivor-conditioned cohort represents dense canopy persistent under these rules, including large parks and wooded patches. It does not represent all street trees, all urban vegetation, tree mortality, or canopy lost before 2024.

### 4.4. Drought Events

Monthly climate records extended from January 1961 through December 2024. On 16 September 2026, we retrieved SPEI from the Google Earth Engine collection CSIC/SPEI/2_11 (SPEIbase v2.11) and retained the native 0.5° grid and six-month accumulation period [21]. SPEI-6 was chosen as the primary exposure because it summarizes seasonal-to-multiseason water-balance deficits relevant to woody vegetation. The six-month timeframe captures seasonal variation without extending to the accumulation period of an entire year. Forest and vegetation responses can nevertheless vary among accumulation scales and climate settings [22,23]. For each month, we calculated the mean over the overlap of the official functional urban area and a 30 km buffer around the configured city coordinate, weighting by the native validity mask and fractional geometric overlap. Values were not interpolated. This regional, FUA-scale exposure describes meteorological conditions rather than local soil or root-zone moisture; it cannot resolve irrigation, soil compaction, artificial water tables, or thermal and moisture gradients within cities. The growing season was April–October in Berlin and Vienna and March–October in Madrid.

A qualifying event comprised at least two consecutive months with SPEI strictly below −1 and at least one month overlapping the configured growing season. Event severity was

*S* = Σ*_t_*(−1 − SPEI*_t_*), for months with SPEI*_t_* < −1. (1)

A multi-year run remained one event. The antecedent was the immediately preceding qualifying SPEI-6 event in the complete 1961–2024 climate record and was never replaced by an earlier event when current-event response support was insufficient. Antecedent identification was therefore independent of HLS availability. The inter-event interval was the number of complete months between the antecedent end and current-event start. Current events still required the predefined HLS observation and outcome-support criterion; climate history was not used to impute missing vegetation responses.

As an additional sensitivity analysis, severity was also summed only across event months within the specified city growing season. The full-run and growing-season-aligned antecedent and current severities were reconstructed directly from the monthly SPEI-6 series. This alignment sensitivity changed the climate predictor, not the event set, HLS outcomes, matching, or support thresholds.

### 4.5. HLS Observations and Quality Control

The response record combined the Earth Engine collections NASA/HLS/HLSL30/v002 and NASA/HLS/HLSS30/v002 during 2013–2024 [24]. L30 begins in April 2013, whereas S30 enters the archived study inventory from 2016. EVI2 was calculated as

EVI2 = 2.5(NIR − red)/(NIR + 2.4red + 1),(2)

following Jiang et al. [13]. NDMI was calculated as

NDMI = (NIR − SWIR1)/(NIR + SWIR1),(3)

using the NIR–SWIR1 NDMI formulation [14]. This follows the normalized-difference approach to vegetation water-sensitive reflectance [15], although Gao’s original NDWI used a different shortwave-infrared wavelength. L30 used B4, B5, and B6; S30 used B4, B8A, and B11. The Earth Engine HLS collections exposed the reflectance bands on the physical scale used by the calculation, so the code applied no additional integer rescaling. Source masks removed native fill; −9999 was introduced only as a local transport sentinel after masking and was never treated as a reflectance. Reflectance was required to be finite and within [0, 1], which preserves the physical units required by the +1 term in EVI2, and NIR + SWIR1 had to be positive. Fmask bits 0–5 had to be zero, explicitly excluding water in bit 5 together with cirrus, cloud, adjacent cloud/shadow, cloud shadow, and snow/ice; aerosol bits 6–7 had to be below 3, excluding high aerosol. The HLS product definition and the original cloud-screening role of Fmask are documented by Ju et al. [24] and Zhu and Woodcock [25], respectively. Valid L30 and S30 observations from overlapping products on the same UTC date were averaged at pixel level. The sensor’s availability and composition changed during the record. Harmonization and same-date averaging did not result in a constant observation frequency or sensor mix. Annual source-inventory unit-day contributions are reported in Supplementary Table S26. These contributions describe availability before pixel-level quality control and are not sensor-specific outcome estimates.

Following the dated reviewer-response protocol, we examined sensor composition from three angles. We first searched selected matched-pair archives for UTC dates on which the same woody pixels had valid observations from both L30 and S30. L30-minus-S30 differences in EVI2 and NDMI were summarized at those pixels without treating pixels as separate drought histories. Next, for the 253 final supported pair-event records, we used the frozen L30 and S30 acquisition inventories for current affected months to derive L30 shares at the unit, pair, and event levels. Because these inventories precede pixel-level quality filtering, their shares describe potential source composition rather than the exact sensor contribution to each retained anomaly. Finally, standardized event-median L30 share was added to each primary event-level regression. Separate L30-only, S30-only, and combined raw month-support screens used the same design-fixed pair denominators and 80% event gate; they are necessary support diagnostics, not sensor-specific outcome reruns.

The original raw denominator contained 618 city-event-mode-year contexts. Independent reconstruction verified 209,854 acquisitions, 118,121 observed dates, 113,323 daily blocks, and 4,752,176 valid woody pixel days. Annual support required at least three clear days per woody pixel. The monthly criterion required observations in at least 60% of the configured growing season months and in every drought-affected growing season month. A pair was considered valid only if both members met the criterion, and an event required at least 80% of its fixed-pair denominator.

### 4.6. Reference Models and Anomalies

Reference models were estimated separately for every event, unit, pixel, and index. The current event calendar year and, when available, the first complete post-event year were omitted. For Berlin, the held-out periods were 2022–2023 and 2023–2024; for Madrid, 2016–2017, 2017–2018, 2019–2020, 2022–2023, and 2023–2024; and for Vienna, 2017–2018, 2018–2020, 2020–2021, 2022–2023, and 2024. This order matches the retained events in Table 2. Holdouts comprised whole years rather than random days. All other available years, including later years not excluded for a given event, contributed to the retrospective training series. We specified three candidate forms in advance: one harmonic without trend, two harmonics without trend, and two harmonics with a linear trend. A fit required at least 30 observed days and eight years containing at least three seasonal observations. Within each index, candidate residuals were compared only on the same held-out pixel-days available to all three models; event-level RMSE was the square root of the unweighted mean squared residual. EVI2 and NDMI were selected independently, without cross-index weighting. Starting with the simplest form, we accepted additional complexity only when it lowered event-level held-out RMSE by more than 0.002 index units. Because current-event observations informed this selection, complexity was not chosen independently of the response years used for anomaly estimation. The fixed one-harmonic no-trend sensitivity removed that decision. Whole-year omission reduced random-fold leakage, but using current-event holdouts for selection and later years for training makes this a retrospective analysis.

The fitted reference estimates seasonal behaviour from the available training observations outside the current event. It should not be read as a drought-free physiological baseline because the training record may contain years affected by other qualifying droughts. We tested whether a reference could instead be fitted after removing every year that overlapped a qualifying event while keeping the predefined minimum of eight training years. Too few years remained in each analytical city, so this stricter model was not fitted, and none of its thresholds were loosened.

Daily anomaly was observed as index minus reference prediction for the same date. The primary yearly anomaly used every observed configured growing-season month in each current-event year, including months outside the qualifying drought run; days were averaged within observed months and months were weighted equally. Missing months were neither imputed nor assigned zero. The affected-month sensitivity restricted aggregation to drought-affected growing-season months and therefore more directly targets the spectral response during drought. For each response year, support was constructed in the following order: persistent woody membership; joint EVI2/NDMI reference support; observation and affected-month coverage; intersection across response years, retaining at least 80% of the woody area in both members of the pair; exact area-weighted unit means; urban minus peri-urban pair differences; and the event median across supported pairs. Current event years were weighted equally within pairs.

### 4.7. Event-Level Association Models

The model form was defined following an outcome-blind audit of event identifiers and the predictor structure. Current severity and duration showed a high correlation (Pearson’s *r* = 0.9478), and the dataset only covered three cities. Given this structure, we did not pursue the hierarchical interaction, random-city, or duration-adjusted models considered earlier.

We estimated two ordinary least squares models for each index:

(1) outcome = intercept + city fixed effects + standardized antecedent severity + standardized current severity;
(2) outcome = intercept + city fixed effects + standardized log(1 + interval) + standardized current severity. Berlin was used as the reference category. Continuous predictors were centred and scaled using the mean and sample standard deviation across the 12 events. Meanwhile, outcomes remained in index units. Events were given equal weighting. We computed OLS coefficients, conventional and HC3 standard errors, and two-sided 95% intervals from Student’s *t* distribution (residual df = 7) with Python 3.13.14 and NumPy 2.5.2 [26]. The estimates were independently reconstructed in statsmodels 0.15.0 using t-based inference. Because five parameters were fitted to 12 observations from three cities, *p*-values and intervals were interpreted as descriptive summaries of uncertainty. Leave-one-event-out and leave-one-city-out analyses assessed stability; neither procedure authorized deleting an observation. HC3 addresses heteroskedasticity and leverage but not serial dependence within a city or dependence among regional droughts. With only three city clusters, cluster-robust inference would be unreliable and was therefore not used. Thus, the event-level observations should not be taken as proof of independence.

### 4.8. Robustness and Sensitivity Analyses

Additional reviewer-driven post hoc robustness analyses followed dated protocols recorded before each added calculation (30 September, 3 October, and 25 October 2026). These records are not a preregistration because the primary results were already known. All specified outputs were retained irrespective of direction or statistical significance. Stability was evaluated with temporal-weighting, affected-month, six-month event-spacing, event- and city-deletion, pair-bootstrap, 30 m inner-buffer, growing-season-aligned severity, fixed one-harmonic reference, and sensor-composition analyses. The fixed-reference analysis recomputed reference support, unit and pair outcomes, event medians, and all focal models. Full L30-only and S30-only outcome reconstructions and alternative-SPEI outcome models remain unperformed and are not presented as completed analyses.

Measurement aggregation uncertainty was evaluated with 10,000 pair-within-event bootstrap replicates and seed 20261003. Supported pairs were resampled with replacement within each retained event; event medians and the four primary models were recalculated. This bootstrap quantifies sensitivity to pair composition conditional on the same 12 retained city-events. It does not resample drought histories or preserve cross-event covariance induced by repeatedly observing the same pairs, and its intervals are not a substitute for exposure-level uncertainty estimates.

A spatial sensitivity analysis transformed the unit polygons to EPSG:3035 and applied a 30 m inward buffer. Eligible woody pixels whose centres fell within the buffered polygon were retained. The exact clipped area weights, reference-pair eligibility flags, and the 0.80 unit and event support rules were preserved. For the climate alignment check, the full-run antecedent and current severities were replaced by values accumulated within each city’s growing season. These replacements were standardized across the same 12 events before the four HC3 models were refitted. Leverage and Cook’s distance were used to summarize influence without removing observations. The supplement reports design-conditional power, exploratory equivalence benchmarks, joint-model checks, and short residual sequences as diagnostic results; none of these was treated as a confirmatory test.

### 4.9. Reproducibility and Audit Trail

Each derived dataset is linked to its inputs via mappings and source hashes. Alongside this, exclusions, variable definitions, missing-value rules, stable codes and import/replay syntax are recorded. The SPSS-compatible workbook stores the adopted analytical inputs and reported outputs. It neither performs the Earth Engine extraction nor documents an executed SPSS analysis. Separate code paths were used to reconstruct the primary analysis and the 30-metre inner-buffer, full-climate antecedent and growing-season-aligned variants. The complete lineage is given in Supplementary Method S1.

### 4.10. Generative-AI Assistance and Author Verification

The authors directed the research, selected the methods, and interpreted the findings. OpenAI Codex using GPT-6.1 Sol (OpenAI, San Francisco, CA, USA; accessed September–October 2026) assisted with literature searches, code preparation and review, computational checks, preparation of figures and tables and Supplementary Materials. Analyses were performed by authors in the project’s computing environments, and the authors verified the sources, code, results, and manuscript content against the underlying data and research records. The authors retained all scientific decision-making authority and responsibility for the final work.

## 5. Conclusions

Across 12 observation-supported recurrent drought events in Berlin, Madrid, and Vienna, the urban-minus-peri-urban spectral contrast of surviving, high-density woody canopy had positive and negative signs, with medians near zero. Estimated associations with antecedent severity and inter-event interval were uncertain. Three out of four primary coefficient signs changed in at least one deletion analysis, and added reference-model and sensor-composition diagnostics did not establish a directional association. The small event sample and seven residual degrees of freedom limited precision and power. This study provides a reproducible matched design and transparent limits on inference. However, it does not establish urban–peri-urban equivalence, physiological drought memory, the absence of a drought-sequence effect, or the health and mortality dynamics of the total urban forest. Broader inference requires more cities and city-event histories, explicit treatment of dependence and observation-based selection, and direct information on species, management, soils, local heat, root-zone moisture, and canopy loss.

## Figures and Tables

**Table 1:** Final matched sample, persistent woody-canopy support, and analytical support.

City	Growing Season	Units	Pairs	Woody Pixels	Area (ha)	Events	Supported/Fixed Pair-Events
Berlin	April–October	60	30	6643	553.09	2	54/60
Madrid	March–October	20	10	867	74.79	5	49/50
Vienna	April–October	60	30	9211	772.08	5	150/150

**Table 2:** Retained current events, drought-sequence attributes, and pair support.

City and Event	Period	Months	Current Severity	Antecedent Severity	Interval (Months)	Supported/Fixed Pairs
Berlin 2022-03	March 2022–February 2023	12	9.318	5.100	13	27/30
Berlin 2023-09	September 2023–October 2023	2	0.629	9.318	6	27/30
Madrid 2016-09	September 2016–January 2017	5	1.048	4.884	7	10/10
Madrid 2017-04	April 2017–February 2018	11	8.098	1.048	2	10/10
Madrid 2018-12	December 2018–October 2019	11	6.616	8.098	9	9/10
Madrid 2022-05	May 2022–November 2022	7	3.891	6.616	30	10/10
Madrid 2023-04	April 2023–September 2023	6	3.298	3.891	4	10/10
Vienna 2017-05	May 2017–August 2017	4	0.601	1.424	19	30/30
Vienna 2018-08	August 2018–April 2019	9	3.531	0.601	11	30/30
Vienna 2020-04	April 2020–May 2020	2	0.826	3.531	11	30/30
Vienna 2022-02	February 2022–December 2022	11	8.698	0.826	20	30/30
Vienna 2024-07	July 2024–August 2024	2	0.344	8.698	18	30/30

**Table 3:** Primary equal-growing-season-month event-level associations. Each model included city fixed effects and standardized current-event severity.

Index	Predictor	Estimate	Model SE	HC3 SE	HC3 95% Interval	*n*	df	Sign Stable in Every Deletion Fit
EVI2	Antecedent severity	−0.00225	0.00245	0.00425	−0.01230 to 0.00781	12	7	No
EVI2	log(1 + inter-event interval)	0.00071	0.00222	0.00189	−0.00377 to 0.00518	12	7	No
NDMI	Antecedent severity	0.00180	0.00356	0.00390	−0.00741 to 0.01102	12	7	Yes
NDMI	log(1 + inter-event interval)	0.00252	0.00299	0.00573	−0.01104 to 0.01608	12	7	No

**Table 4:** Sensitivity analysis requiring at least six months between events. Predictors were standardized again in the ten-event sample; outcome units were unchanged.

Index	Predictor	Estimate	HC3 95% Interval	*n*	df
EVI2	Antecedent severity	−0.00209	−0.01700 to 0.01281	10	5
EVI2	log(1 + inter-event interval)	0.00345	−0.00893 to 0.01583	10	5
NDMI	Antecedent severity	0.00331	−0.00919 to 0.01581	10	5
NDMI	log(1 + inter-event interval)	0.00817	−0.01636 to 0.03269	10	5

**Table 5:** Primary estimates and robustness analyses; values in parentheses are 95% intervals. In the bootstrap column, the point estimate is the mean focal coefficient over 10,000 pair-within-event resamples, and the interval spans the 2.5th–97.5th percentiles. As this is an average across resamples, it may differ from the coefficient based on the original event medians. HC3 standard errors were used for the remaining intervals. In the season-aligned models, full-run severity was replaced by severity accumulated during the specified city’s growing season. The other columns contain fitted-model coefficients and HC3 intervals. Coefficients are expressed in index units for a one-standard-deviation increase in the stated predictor; interval models use log(1 + interval). Cook’s flags are the number of events exceeding 4/*n* in the primary model.

Index	Predictor	Primary, HC3	Pair Bootstrap	30 m Inner Buffer	Season-Aligned Severity	Cook Flags
EVI2	Antecedent severity	−0.00225 (−0.01230 to 0.00781)	−0.00149 (−0.00497 to 0.00263)	−0.00249 (−0.01258 to 0.00760)	−0.00113 (−0.00922 to 0.00696)	3
EVI2	Inter-event interval	0.00071 (−0.00377 to 0.00518)	0.00122 (−0.00132 to 0.00421)	0.00072 (−0.00455 to 0.00600)	0.00088 (−0.00332 to 0.00507)	2
NDMI	Antecedent severity	0.00180 (−0.00741 to 0.01102)	0.00142 (−0.00454 to 0.00615)	0.00214 (−0.00812 to 0.01239)	0.00311 (−0.00609 to 0.01232)	1
NDMI	Inter-event interval	0.00252 (−0.01104 to 0.01608)	0.00201 (−0.00360 to 0.00675)	0.00256 (−0.01132 to 0.01643)	0.00241 (−0.01239 to 0.01721)	2

## Data Availability

The journal’s Supplementary Materials consist of Supplementary Methods S1, Tables S1–S26, and Figures S1–S3. A separate versioned data, code, and provenance archive supporting the study is publicly available on Zenodo at https://doi.org/10.5281/zenodo.23297392. The Zenodo archive contains the derived analytical datasets; machine-readable source tables and figure files corresponding to the reported supplementary materials; variable definitions; missing-value conventions; stable identifiers and categorical codes; input-to-output mappings; analysis protocols; Google Earth Engine extraction and configuration code; analysis code; environment specifications; and SPSS-compatible import and replay materials. The Zenodo archive is an external reproducibility resource and is not part of the journal’s Supplementary Materials. Source satellite, land-cover, settlement, boundary, elevation, and climate products remain available from their respective providers under the providers’ access and citation terms and are not redistributed. The Excel/SPSS-compatible workbooks begin with derived analytical tables and do not reproduce the Google Earth Engine extraction; the supplied SPSS syntax was not executed in native SPSS.

## Supplementary Materials

The following supporting information can be downloaded at: https://www.mdpi.com/article/10.3390/plants15213401/s1. Supplementary Methods S1: Audit and Extended Analyses; Table S1: Pre- and post-match balance by city and stratum; Table S2: Matched sample summary; Table S3: Primary-model event influence records; Table S4: Pair-level outcome distribution summaries; Table S5: Event-predictor correlations; Table S6: Affected-month event outcomes; Table S7: Alternative temporal-weighting models; Table S8: Current and post-event descriptive summaries; Table S9: Six-month event-spacing inclusion record; Table S10: Drought-year-free reference feasibility; Table S11: Design-conditional precision and equivalence benchmark; Table S12: Pair-within-event bootstrap model summaries; Table S13: Joint antecedent-severity and interval diagnostics; Table S14: Event influence summary; Table S15: Reference-model selection audit; Table S16: Persistent-canopy survivor-conditioning summary; Table S17: Expanded matching and distance diagnostics; Table S17B: Fixed matched-pair geographic distances and built-fraction descriptors; Table S18: SPEI accumulation-scale event inventory; Table S19: SPEI event-overlap diagnostics; Table S20: Descriptive residual temporal-dependence summaries; Table S21: Thirty-metre inner-buffer event outcomes; Table S22: Thirty-metre inner-buffer model coefficients; Table S23: Full-climate antecedent audit; Table S24: Growing-season-aligned event predictors; Table S25: Growing-season-aligned model results; Table S26A: Annual HLS source-inventory availability across all 140 fixed analytical units before pixel-level quality control; Table S26B: Annual HLS source-inventory availability by analytical city before pixel-level quality control; Figure S1: Supported-pair distributions; Figure S2: Pair-level outcome distributions; Figure S3: Climate-only SPEI-scale event inventory.

## Abbreviations

The following abbreviations are used in this manuscript:

EVI2	two-band enhanced vegetation index
FUA	Functional Urban Area
GHSL	Global Human Settlement Layer
HLS	Harmonized Landsat–Sentinel-2
GenAI	Generative Artificial Intelligence
L30	Landsat-derived HLS product
NDMI	normalized difference moisture index
RMSE	root mean square error
S30	Sentinel-2-derived HLS product
SPEI	Standardized Precipitation Evapotranspiration Index
