# Supplementary material for: Spectral Responses of Urban and Peri-Urban Woody Canopy to Recurrent Drought: A Matched Three-City Pilot
Source: Plants (Basel). 2026 Nov 2;15(21):3401. doi: 10.3390/plants15213401 (PMC13765884; doi:10.3390/plants15213401)
Supplement: Supplementary file 1 [file plants-15-03401-s001.zip › Supplementary Methods S1.pdf]

Supplementary Methods S1

Audit and Extended Analyses

Spectral Responses of Urban and Peri-Urban Woody Canopy to Recurrent Drought: A Matched Three-City Pilot

S1. Scope and version control

This supplement documents the audit logic, extended analyses, and diagnostic workflow supporting the manuscript. Earlier manuscript releases were preserved as immutable predecessors. The integrated revision used already downloaded and independently audited project data, added no city, and did not re-download raw satellite observations. Its new analyses were defined in dated reviewer-driven post hoc protocols before their results were calculated; the protocols are not preregistrations because the primary results were already known.

S2. Raw extraction and independent audit

The original combined-HLS denominator contained 618 city-event-mode-year contexts. The completed extraction retained every defined context, including failed cells and unsupported outcomes. An independent implementation verified source inventories, the persistent woody mask, serialized processing graphs, daily arrays, coordinate grids, observation counts, and the absence of valid observations outside the woody mask. Preparation, synthetic checks, and offline validation were not treated as empirical results.

S3. Reference, outcome, and denominator audit

All candidate reference fits were reconstructed independently before the fixed selection rule was applied. Outcome reconstruction verified pixel-year, unit-year, pair-event, and event-level tables, including missing outcomes and recorded exclusion reasons. Common footprints, exact clipped-area weights, pair support, and event support were recalculated without reading fitted effect directions. Failed cells remained in the denominator, and no city, event, pair, or observation-quality threshold was selected using coefficient direction or statistical significance.

S4. Primary and targeted sensitivity models

The primary event-level models used 12 retained drought events in Berlin, Madrid, and Vienna. City fixed effects and HC3 covariance estimation were retained from the audited analysis. Primary coefficients, diagnostics, leave-one-event and leave-one-city fits, affected-month robustness, and the six-month event-spacing sensitivity were reconstructed through an independent code path. The drought-year-free reference exercise was a structural feasibility check: no model was fitted where fewer than eight non-drought candidate years remained.

S5. Full-climate antecedent audit

Antecedent droughts were reconstructed as the immediately preceding qualifying SPEI-6 runs in the complete saved 1961–2024 climate record. Antecedent identification did not require HLS vegetation observations because antecedent severity and inter-event interval are climate predictors. All 26 fixed current-event candidates had an identifiable antecedent. The unchanged 80% HLS observation-support gate retained 12 current events and excluded 14. Five attrition labels changed from an HLS-era antecedent limitation to insufficient response support, but no analytical event, outcome, pair, threshold, or fitted primary result changed.

S6. Growing-season-aligned severity sensitivity

For each retained current event and its antecedent, full-run SPEI-6 severity was reconstructed from monthly values and then partitioned into fixed growing-season and dormant-season components. Growing seasons were March–October for Madrid and April–October for Berlin and Vienna. Every drought-run month with SPEI-6 below −1 and falling within the corresponding calendar-month window contributed `−1 − SPEI-6` to aligned severity. Full-run reconstruction agreed with the frozen event catalogue, and the aligned component independently agreed with its saved growing-season deficit field.

Aligned antecedent and current severities were standardized across the unchanged 12 events. Four OLS models with city fixed effects, equal event weights, and HC3 covariance were fitted: two index-specific antecedent models adjusted for aligned current severity, and two interval models adjusted for aligned current severity. All four focal coefficient directions matched the primary models and every 95% interval included zero. This is a response-window-alignment sensitivity, not a new primary drought definition or evidence of physiological memory.

S7. Design-conditional precision and equivalence benchmark

For each focal coefficient, the design-conditional 80% minimum detectable coefficient used the fitted design matrix, residual variance, two-sided alpha of 0.05, and the noncentral t distribution. Two one-sided tests were also calculated against a reviewer-proposed margin of ±0.01 index units. This margin was not validated as a biological threshold. The calculations are retained as supplementary numerical diagnostics and are not used to claim biological equivalence or absence.

S8. Pair-within-event measurement bootstrap

The measurement bootstrap resampled matched pairs with replacement within each retained event, recalculated area-weighted event contrasts, and refitted the unchanged event-level models. It used 10,000 replicates and seed 20261003. Percentile intervals describe uncertainty from pair aggregation conditional on the same 12 city-event histories. Bootstrap replicates were not counted as new drought histories and were not used to replace event-level inference.

S9. Joint, influence, and residual diagnostics

Joint diagnostic models entered antecedent drought severity and log-transformed inter-event interval together with city fixed effects. These fits had six residual degrees of freedom and were interpreted as diagnostics. Event influence records included leverage, Cook's distance, DFBETAs, and model changes after deleting one event. Threshold exceedances were retained rather than removed. Within-city residual lag-1 correlation and Durbin–Watson statistics were saved only as descriptive summaries because Berlin contained two events and Madrid and Vienna contained five events each.

S10. Matching and spatial diagnostics

Matched-pair centroid distances and expanded balance diagnostics were reported for the fixed specification. Built fraction operationalized the intended urban–peri-urban settlement contrast and was therefore retained as a descriptor rather than forced into the balance target. Geographic distance was not an optimization term. Distance, balance, and built-fraction diagnostics were interpreted as design limitations rather than causal-balance certification; no outcome-driven rematching was performed.

S11. Persistent-canopy survivor conditioning

Prospective baseline woody area, persistent analytical area, and cumulative mapped-loss overlap were summarized by city and stratum. Retained-area fractions quantify the cohort restriction created by the persistent-canopy design. Mapped loss was not attributed to drought, and the analysis does not estimate mortality or total urban-forest loss.

S12. SPEI accumulation-scale inventory

The frozen climate-only drought-run rule was applied separately to SPEI-3, SPEI-6, and SPEI-12 over 1961–2024 to quantify how accumulation scale changed the qualifying-event inventory. Event-overlap tables were saved. Vegetation outcomes, reference models, support gates, and common footprints were not relabelled or recomputed for SPEI-3 or SPEI-12; consequently, these inventories are definition diagnostics rather than alternative vegetation-effect analyses.

S13. Thirty-metre inner-buffer sensitivity

The protocol-defined spatial sensitivity applied a 30 m inward buffer to fixed analysis units while preserving every scientific threshold, event definition, reference model, persistent-canopy rule, and support gate. The geometry-stage audit covered both unit members of all 260 fixed pair-event records (520 unit-event cases) and retained 511. Outcome reconstruction then rejoined the 259 retrievable pair-event records before applying the unchanged pair and event gates; all 12 events were retained. Event outcomes and HC3 model coefficients were independently reconstructed. No focal sign changed and every interval included zero. The buffer analysis reduces concern about immediate unit edges but does not establish the absence of spatial mixing or geolocation error.

S14. Software and interpretation limits

Earth Engine performed satellite extraction. Python produced the independently audited derived tables, models, figures, and Word source. Excel files were authored as interoperability artifacts. The analysis is observational and event-level; pixels, units, pairs, and bootstrap replicates improve measurement but are not additional independent drought histories. The manuscript does not claim a causal urban effect, physiological drought memory, biological equivalence, a representative European effect, or a confirmatory null result.

S15. Ordered two-index common-footprint construction

For every event, unit, response year, and pixel, the outcome implementation applied the following order: (1) fixed persistent-woody membership; (2) joint EVI2 and NDMI reference-model support; (3) observed-day and affected-month coverage; (4) intersection across current-event response years; (5) retention only where at least 80% of fixed woody area remained in both matched units; (6) exact clipped-area weighting within each unit; (7) urban-minus-peri-urban pair differencing; and (8) the median across supported pairs. The primary outcome used all observed configured growing-season months in current-event years. The affected-month sensitivity restricted temporal aggregation to event months overlapping the growing season.

S16. Annual HLS source-inventory contribution diagnostic

Table S26 reports source-product availability from all 1,680 immutable analytical unit-year inventories. Its two panels provide annual totals across all fixed analytical units (S26A) and the corresponding city-by-year detail (S26B). A unit-date was classified as L30-only, S30-only, or both when at least one archived source asset from the corresponding product was present on that UTC date. These counts precede pixel-level Fmask, aerosol, physical-reflectance, and persistent-woody validity checks; they quantify changing source availability, not valid pixel-days, sensor-specific vegetation outcomes, or independent observations. The audit read 1,680 of 1,680 authoritative inventories and found no product, year, or observed-day invariant failure.
