# Supplementary material for: Spectral Responses of Urban and Peri-Urban Woody Canopy to Recurrent Drought: A Matched Three-City Pilot
Source: Plants (Basel). 2026 Nov 2;15(21):3401. doi: 10.3390/plants15213401 (PMC13765884; doi:10.3390/plants15213401)
Supplement: Supplementary file 1 [file plants-15-03401-s001.zip › Supplementary Materials.pdf]

Supplementary Materials

Spectral Responses of Urban and Peri-Urban Woody Canopy to Recurrent Drought: A Matched Three-City Pilot

Manuscript ID: plants-4656158

This file contains Tables S1-S26 and Figures S1-S3 cited in the manuscript. Blank cells retain unavailable or not-applicable values from the audited source tables. Numeric values are displayed to six significant digits; no analytical values were recalculated for presentation.

Table S1. Pre- and post-match balance by city and stratum.

City	Variable	Stage	Urban Mean	Peri Mean	Smd Fixed Candidate Sd	Smd Stage Specific Sd Diagnostic
Berlin	background mean elevation m	candidate	43.0159	44.8489	-0.201211	-0.201211
Berlin	background mean elevation m	balanced	42.0943	41.9968	0.0106985	0.0104612
Berlin	clim mean temperature c 1981 2010	candidate	9.6994	9.47946	1.25637	1.25637
Berlin	clim mean temperature c 1981 2010	balanced	9.50187	9.50494	-0.0175078	-0.0252041
Berlin	clim annual precipitation mm 1981 2010	candidate	578.633	579.002	-0.0229314	-0.0229314
Berlin	clim annual precipitation mm 1981 2010	balanced	575.357	575.318	0.00241405	0.00217537
Berlin	retained woody area ha approx	candidate	13.8244	22.6177	-0.518335	-0.518335
Berlin	retained woody area ha approx	balanced	9.08871	9.33769	-0.014677	-0.0268437
Madrid	background mean elevation m	candidate	648.314	684.249	-0.46102	-0.46102
Madrid	background mean elevation m	balanced	650.247	642.608	0.0979955	0.162103
Madrid	clim mean temperature c 1981 2010	candidate	14.7681	14.2404	1.00775	1.00775
Madrid	clim mean temperature c 1981 2010	balanced	14.6184	14.575	0.0830321	0.242709
Madrid	clim annual precipitation mm 1981 2010	candidate	402.12	398.525	0.238258	0.238258
Madrid	clim annual precipitation mm 1981 2010	balanced	401.287	400.467	0.0543513	0.0590029
Madrid	retained woody area ha approx	candidate	4.87816	3.68847	0.357296	0.357296
Madrid	retained woody area ha approx	balanced	3.89718	3.582	0.0946564	0.192988
Vienna	background mean elevation m	candidate	211.6	246.287	-0.62114	-0.62114
Vienna	background mean elevation m	balanced	214.374	219.224	-0.0868529	-0.088962
Vienna	clim mean temperature c 1981 2010	candidate	10.3382	10.0297	0.876365	0.876365
Vienna	clim mean temperature c 1981 2010	balanced	10.2393	10.2043	0.0995002	0.123932
Vienna	clim annual precipitation mm 1981 2010	candidate	593.132	598.937	-0.265205	-0.265205
Vienna	clim annual precipitation mm 1981 2010	balanced	592.28	591.726	0.0253305	0.02956
Vienna	retained woody area ha approx	candidate	10.7678	27.9907	-1.04682	-1.04682
Vienna	retained woody area ha approx	balanced	12.1078	13.626	-0.092271	-0.142163

Table S2. Matched sample summary.

City	Selected Pairs	Passes Count Rule	Solver Status	Max Abs Smd Fixed Candidate Sd
Berlin	30	Yes	0	0.0175078
Madrid	10	Yes	0	0.0979955
Vienna	30	Yes	0	0.0995002

Table S3. Primary-model event influence records. *Column panel 1 of 3; identifier columns are repeated.*

Index	Predictor	N	Residual df	Condition Number	Estimate	HC3 CI Low	HC3 CI High
EVI2	antecedent	12	7	6.29139	-0.00224701	-0.0122998	0.00780578
EVI2	interval	12	7	5.28647	0.000705117	-0.0037659	0.00517614
NDMI	antecedent	12	7	6.29139	0.00180339	-0.00741421	0.011021
NDMI	interval	12	7	5.28647	0.00251918	-0.0110382	0.0160765

Table S3. Primary-model event influence records. *Column panel 2 of 3; identifier columns are repeated.*

Index	Predictor	Maximum Leverage	Maximum Absolute Studentized Residual	Maximum Cook Distance	Leave-one-event Minimum	Leave-one-event Maximum	Leave-one-city Minimum
EVI2	antecedent	0.659578	1.91266	0.741449	-0.00416442	0.00119461	-0.00608798
EVI2	interval	0.695701	2.03183	0.665639	-0.000117037	0.00171751	-0.000117037
NDMI	antecedent	0.659578	1.88981	0.375728	0.000155656	0.00474092	0.00143182
NDMI	interval	0.695701	2.01442	0.926872	-0.00288667	0.00426025	-0.000513429

Table S3. Primary-model event influence records. *Column panel 3 of 3; identifier columns are repeated.*

Index	Predictor	Leave-one-city Maximum	All Leave-outs Preserve Sign
EVI2	antecedent	0.00141421	No
EVI2	interval	0.00376132	No
NDMI	antecedent	0.00223073	Yes
NDMI	interval	0.0028586	No

Table S4. Pair-level outcome distribution summaries. *Column panel 1 of 2; identifier columns are repeated.*

City	Event ID	Vegetation Index	N Supported Pairs	Median	Q1	Q3	Minimum
Berlin	Berlin S06 2022-03	EVI2	27	0.000843794	-0.00240881	0.011375	-0.0223521
Berlin	Berlin S06 2022-03	NDMI	27	-0.000112929	-0.00440735	0.00701141	-0.0172
Berlin	Berlin S06 2023-09	EVI2	27	-0.00290778	-0.0146834	0.00565723	-0.0308287
Berlin	Berlin S06 2023-09	NDMI	27	-0.00110712	-0.0139548	0.00356617	-0.0243552
Madrid	Madrid S06 2016-09	EVI2	10	0.00873104	-0.00551102	0.0163887	-0.0312851
Madrid	Madrid S06 2016-09	NDMI	10	-0.0105392	-0.0271448	0.0159327	-0.038162
Madrid	Madrid S06 2017-04	EVI2	10	-0.00181707	-0.0112106	0.00487492	-0.0224617
Madrid	Madrid S06 2017-04	NDMI	10	0.000706742	-0.00489485	0.0079133	-0.0271617
Madrid	Madrid S06 2018-12	EVI2	9	-0.00954836	-0.0166579	0.00513181	-0.0206768
Madrid	Madrid S06 2018-12	NDMI	9	0.000601772	-0.010151	0.0109153	-0.0455159
Madrid	Madrid S06 2022-05	EVI2	10	0.00549088	0.00283889	0.0114471	-0.00761273
Madrid	Madrid S06 2022-05	NDMI	10	0.0163639	-0.00223611	0.0268544	-0.0188662
Madrid	Madrid S06 2023-04	EVI2	10	0.0113255	-0.00235936	0.018148	-0.0179334
Madrid	Madrid S06 2023-04	NDMI	10	0.016602	-0.00646288	0.0376242	-0.0260417
Vienna	Vienna S06 2017-05	EVI2	30	9.8546e-05	-0.00424386	0.00887297	-0.0685087
Vienna	Vienna S06 2017-05	NDMI	30	-0.00347139	-0.0122946	0.00845294	-0.0733197
Vienna	Vienna S06 2018-08	EVI2	30	-0.00557183	-0.0125898	0.00374913	-0.0404151
Vienna	Vienna S06 2018-08	NDMI	30	-0.00163757	-0.00608104	0.00547475	-0.0591041
Vienna	Vienna S06 2020-04	EVI2	30	0.00328693	-0.00806321	0.0106756	-0.0176981
Vienna	Vienna S06 2020-04	NDMI	30	0.00161778	-0.00774475	0.00914416	-0.0327703
Vienna	Vienna S06 2022-02	EVI2	30	-0.000951718	-0.00932381	0.0129361	-0.0450912
Vienna	Vienna S06 2022-02	NDMI	30	0.00316973	-0.00628061	0.0118517	-0.0403189
Vienna	Vienna S06 2024-07	EVI2	30	0.00117658	-0.00508295	0.0111511	-0.0238906
Vienna	Vienna S06 2024-07	NDMI	30	0.0018127	-0.00545989	0.010315	-0.0237162

Table S4. Pair-level outcome distribution summaries. *Column panel 2 of 2; identifier columns are repeated.*

City	Event ID	Vegetation Index	Maximum	Proportion Above Zero	Proportion Equal Zero
Berlin	Berlin S06 2022-03	EVI2	0.0401451	0.555556	0
Berlin	Berlin S06 2022-03	NDMI	0.0417639	0.481481	0
Berlin	Berlin S06 2023-09	EVI2	0.0180144	0.37037	0
Berlin	Berlin S06 2023-09	NDMI	0.0459353	0.407407	0
Madrid	Madrid S06 2016-09	EVI2	0.0319865	0.6	0
Madrid	Madrid S06 2016-09	NDMI	0.0631823	0.4	0
Madrid	Madrid S06 2017-04	EVI2	0.0160415	0.4	0
Madrid	Madrid S06 2017-04	NDMI	0.0398174	0.5	0
Madrid	Madrid S06 2018-12	EVI2	0.0118744	0.444444	0
Madrid	Madrid S06 2018-12	NDMI	0.0308089	0.555556	0
Madrid	Madrid S06 2022-05	EVI2	0.0261682	0.8	0
Madrid	Madrid S06 2022-05	NDMI	0.0295938	0.6	0
Madrid	Madrid S06 2023-04	EVI2	0.0392811	0.6	0
Madrid	Madrid S06 2023-04	NDMI	0.080417	0.7	0
Vienna	Vienna S06 2017-05	EVI2	0.0210852	0.533333	0
Vienna	Vienna S06 2017-05	NDMI	0.0236766	0.4	0
Vienna	Vienna S06 2018-08	EVI2	0.0139498	0.4	0
Vienna	Vienna S06 2018-08	NDMI	0.0158686	0.466667	0
Vienna	Vienna S06 2020-04	EVI2	0.0426912	0.533333	0
Vienna	Vienna S06 2020-04	NDMI	0.0351218	0.533333	0
Vienna	Vienna S06 2022-02	EVI2	0.0378424	0.466667	0
Vienna	Vienna S06 2022-02	NDMI	0.034278	0.6	0
Vienna	Vienna S06 2024-07	EVI2	0.0287432	0.6	0
Vienna	Vienna S06 2024-07	NDMI	0.0310057	0.566667	0

Table S5. Event-predictor correlations.

Method	Row Variable	Column Variable	Correlation	N Events
pearson	antecedent severity	antecedent severity	1	12
pearson	antecedent severity	log1p interval	0.100264	12
pearson	antecedent severity	current severity	-0.300905	12
pearson	antecedent severity	duration months	-0.370632	12
pearson	log1p interval	antecedent severity	0.100264	12
pearson	log1p interval	log1p interval	1	12
pearson	log1p interval	current severity	-0.10659	12
pearson	log1p interval	duration months	-0.100305	12
pearson	current severity	antecedent severity	-0.300905	12
pearson	current severity	log1p interval	-0.10659	12
pearson	current severity	current severity	1	12
pearson	current severity	duration months	0.947794	12
pearson	duration months	antecedent severity	-0.370632	12
pearson	duration months	log1p interval	-0.100305	12
pearson	duration months	current severity	0.947794	12
pearson	duration months	duration months	1	12
spearman	antecedent severity	antecedent severity	1	12
spearman	antecedent severity	log1p interval	-0.0420316	12
spearman	antecedent severity	current severity	-0.307692	12
spearman	antecedent severity	duration months	-0.333367	12
spearman	log1p interval	antecedent severity	-0.0420316	12
spearman	log1p interval	log1p interval	1	12
spearman	log1p interval	current severity	0.0175132	12
spearman	log1p interval	duration months	0.0284214	12
spearman	current severity	antecedent severity	-0.307692	12
spearman	current severity	log1p interval	0.0175132	12
spearman	current severity	current severity	1	12
spearman	current severity	duration months	0.95045	12
spearman	duration months	antecedent severity	-0.333367	12
spearman	duration months	log1p interval	0.0284214	12
spearman	duration months	current severity	0.95045	12
spearman	duration months	duration months	1	12

Table S6. Affected-month event outcomes. *Column panel 1 of 2; identifier columns are repeated.*

City	Event ID	Fixed Candidate Pairs	Supported Pairs	Supported Pair Fraction	Median Affected Month Anomaly EVI2 Urban	Median Affected Month Anomaly EVI2 Peri	Median Affected Month Anomaly EVI2 Urban Minus Peri
Berlin	Berlin S06 2022-03	30	27	0.9	-0.00569692	-0.00823601	0.000843794
Berlin	Berlin S06 2023-09	30	27	0.9	0.00604255	0.0118742	-0.0048789
Madrid	Madrid S06 2016-09	10	10	1	-0.00547254	-0.0104349	0.00408924
Madrid	Madrid S06 2017-04	10	10	1	-0.0166901	-0.0107156	-0.00521951
Madrid	Madrid S06 2018-12	9	9	1	-0.00288956	0.00616752	-0.00954836
Madrid	Madrid S06 2022-05	10	10	1	0.00509247	-0.00396657	0.00699836
Madrid	Madrid S06 2023-04	10	10	1	-0.000461128	-0.0125676	0.0157229
Vienna	Vienna S06 2017-05	30	30	1	-0.00214292	-0.00305249	-0.000515792
Vienna	Vienna S06 2018-08	30	30	1	-0.00758424	-0.00881524	-0.000457342
Vienna	Vienna S06 2020-04	30	30	1	0.00723005	0.00261889	0.00236563
Vienna	Vienna S06 2022-02	30	30	1	-0.0186231	-0.0196474	-0.000951718
Vienna	Vienna S06 2024-07	30	30	1	-0.0287895	-0.0375041	0.00497909

Table S6. Affected-month event outcomes. *Column panel 2 of 2; identifier columns are repeated.*

City	Event ID	Median Affected Month Anomaly NDMI Urban	Median Affected Month Anomaly NDMI Peri	Median Affected Month Anomaly NDMI Urban Minus Peri
Berlin	Berlin S06 2022-03	-0.0156306	-0.014291	-0.000112929
Berlin	Berlin S06 2023-09	0.0163243	0.0184256	-0.00119039
Madrid	Madrid S06 2016-09	0.00158047	0.00433206	-0.0103297
Madrid	Madrid S06 2017-04	-0.016925	-0.0196735	0.00185799
Madrid	Madrid S06 2018-12	0.00572436	0.00761524	0.000601772
Madrid	Madrid S06 2022-05	-0.0110609	-0.0194121	0.0124255
Madrid	Madrid S06 2023-04	-0.0171571	-0.0333026	0.0236143
Vienna	Vienna S06 2017-05	-0.00437725	9.0938e-05	-0.00238793
Vienna	Vienna S06 2018-08	-0.0111642	-0.0102176	0.00146233
Vienna	Vienna S06 2020-04	-0.00596256	-0.0158329	0.00760426
Vienna	Vienna S06 2022-02	-0.0225451	-0.0256605	0.00316973
Vienna	Vienna S06 2024-07	0.000982846	-0.00203192	0.00347939

Table S7. Alternative temporal-weighting models.

Weighting	Index	Predictor	Estimate	HC3 95% CI	Leave-out Sign Stable
equal month	EVI2	Antecedent severity (1 SD)	-0.00225	-0.01230 to 0.00781	No
equal month	EVI2	log1p interval (1 SD)	0.00071	-0.00377 to 0.00518	No
equal month	NDMI	Antecedent severity (1 SD)	0.0018	-0.00741 to 0.01102	Yes
equal month	NDMI	log1p interval (1 SD)	0.00252	-0.01104 to 0.01608	No
equal valid day	EVI2	Antecedent severity (1 SD)	-0.00227	-0.01323 to 0.00868	No
equal valid day	EVI2	log1p interval (1 SD)	0.00097	-0.00458 to 0.00652	No
equal valid day	NDMI	Antecedent severity (1 SD)	0.00169	-0.00704 to 0.01041	No
equal valid day	NDMI	log1p interval (1 SD)	0.00262	-0.01015 to 0.01539	No
acquisition weighted	EVI2	Antecedent severity (1 SD)	-0.00227	-0.01399 to 0.00945	No
acquisition weighted	EVI2	log1p interval (1 SD)	0.00133	-0.00430 to 0.00697	Yes
acquisition weighted	NDMI	Antecedent severity (1 SD)	0.0018	-0.00698 to 0.01058	No
acquisition weighted	NDMI	log1p interval (1 SD)	0.00272	-0.01016 to 0.01560	No

Table S8. Current and post-event descriptive summaries. *Column panel 1 of 2; identifier columns are repeated.*

City	Event ID	Post Year	Fixed Candidate Pairs	Supported Pairs	Median Equal Month Anomaly EVI2 Urban Minus Peri Current	Median Equal Month Anomaly EVI2 Urban Minus Peri Post	Median Equal Month Anomaly EVI2 Urban Minus Peri Post Minus Current
Berlin	Berlin S06 2023-09	2024	30	27	-0.00290778	0.000618249	0.00419118
Madrid	Madrid S06 2017-04	2018	10	10	-0.00181707	-0.00823078	-0.0101573
Madrid	Madrid S06 2018-12	2020	9	9	-0.00954836	-0.0043222	0.00124652
Madrid	Madrid S06 2023-04	2024	10	10	0.0113255	0.012256	-0.00613157
Vienna	Vienna S06 2020-04	2021	30	30	0.00328693	0.0022067	-0.000781957
Vienna	Vienna S06 2022-02	2023	30	30	-0.000951718	0.00666285	0.00372564

Table S8. Current and post-event descriptive summaries. *Column panel 2 of 2; identifier columns are repeated.*

City	Event ID	Median Equal Month Anomaly NDMI Urban Minus Peri Current	Median Equal Month Anomaly NDMI Urban Minus Peri Post	Median Equal Month Anomaly NDMI Urban Minus Peri Post Minus Current
Berlin	Berlin S06 2023-09	-0.00110712	-0.00111935	0.00189254
Madrid	Madrid S06 2017-04	0.000706742	-0.0186528	-0.0221535
Madrid	Madrid S06 2018-12	0.000601772	-0.0115655	-0.00709914
Madrid	Madrid S06 2023-04	0.016602	0.00145929	-0.0256349
Vienna	Vienna S06 2020-04	0.00161778	0.00302641	-0.000816167
Vienna	Vienna S06 2022-02	0.00316973	0.00704088	0.00528471

Table S9. Six-month event-spacing inclusion record.

City	Event ID	Antecedent Event ID	Drought Free Interval Months	Minimum Required Months	Retained In Six Month Sensitivity	Exclusion Reason
Berlin	Berlin S06 2022-03	Berlin S06 2020-04	13	6	Yes	
Berlin	Berlin S06 2023-09	Berlin S06 2022-03	6	6	Yes	
Madrid	Madrid S06 2016-09	Madrid S06 2015-05	7	6	Yes	
Madrid	Madrid S06 2017-04	Madrid S06 2016-09	2	6	No	interval shorter than spei accumulation window
Madrid	Madrid S06 2018-12	Madrid S06 2017-04	9	6	Yes	
Madrid	Madrid S06 2022-05	Madrid S06 2018-12	30	6	Yes	
Madrid	Madrid S06 2023-04	Madrid S06 2022-05	4	6	No	interval shorter than spei accumulation window
Vienna	Vienna S06 2017-05	Vienna S06 2015-07	19	6	Yes	
Vienna	Vienna S06 2018-08	Vienna S06 2017-05	11	6	Yes	
Vienna	Vienna S06 2020-04	Vienna S06 2018-08	11	6	Yes	
Vienna	Vienna S06 2022-02	Vienna S06 2020-04	20	6	Yes	
Vienna	Vienna S06 2024-07	Vienna S06 2022-02	18	6	Yes	

Table S10. Drought-year-free reference feasibility.

City	Hls Record Years	All Qualifying Drought Years	Remaining Candidate Training Years	Remaining Calendar Year Count	Minimum Required Training Years	Drought Year Free Reference Structurally Feasible	Decision
Berlin	2013;2014;2015;2016;2017;2018;2019;2020;2021;2022;2023;2024	2014;2015;2016;2017;2018;2019;2020;2021;2022;2023	2013;2024	2	8	No	not fitted insufficient calendar years
Hamburg	2013;2014;2015;2016;2017;2018;2019;2020;2021;2022;2023;2024	2014;2016;2017	2013;2015;2018;2019;2020;2021;2022;2023;2024	9	8	Yes	eligible for pixel level fit
Helsinki	2013;2014;2015;2016;2017;2018;2019;2020;2021;2022;2023;2024	2018;2019;2022	2013;2014;2015;2016;2017;2020;2021;2023;2024	9	8	Yes	eligible for pixel level fit
Madrid	2013;2014;2015;2016;2017;2018;2019;2020;2021;2022;2023;2024	2014;2015;2016;2017;2018;2019;2022;2023	2013;2020;2021;2024	4	8	No	not fitted insufficient calendar years
Vienna	2013;2014;2015;2016;2017;2018;2019;2020;2021;2022;2023;2024	2014;2015;2017;2018;2019;2020;2022;2024	2013;2016;2021;2023	4	8	No	not fitted insufficient calendar years

Table S11. Design-conditional precision and equivalence benchmark. *Column panel 1 of 4; identifier columns are repeated.*

Method	Vegetation Index	Model ID	Focal Term	N Events	Residual df	Ols Residual Sigma	Focal Estimate
equal month	EVI2	antecedent	z antecedent severity	12	7	0.00605976	-0.00224701
equal month	EVI2	interval	z log1p interval	12	7	0.00636901	0.000705117
equal month	NDMI	antecedent	z antecedent severity	12	7	0.00883092	0.00180339
equal month	NDMI	interval	z log1p interval	12	7	0.00856633	0.00251918

Table S11. Design-conditional precision and equivalence benchmark. *Column panel 2 of 4; identifier columns are repeated.*

Method	Vegetation Index	Model ID	Focal Term	HC3 Se	Equivalence Margin Absolute Index Units	HC3 90% CI Low	HC3 90% CI High
equal month	EVI2	antecedent	z antecedent severity	0.00425133	0.01	-0.0103015	0.00580746
equal month	EVI2	interval	z log1p interval	0.00189079	0.01	-0.00287714	0.00428738
equal month	NDMI	antecedent	z antecedent severity	0.00389812	0.01	-0.00558192	0.00918869
equal month	NDMI	interval	z log1p interval	0.0057334	0.01	-0.00834319	0.0133816

Table S11. Design-conditional precision and equivalence benchmark. *Column panel 3 of 4; identifier columns are repeated.*

Method	Vegetation Index	Model ID	Focal Term	Tost P Lower	Tost P Upper	Equivalent At Alpha 0 05	Two Sided Power For Absolute 0 01
equal month	EVI2	antecedent	z antecedent severity	0.0554857	0.0118131	No	0.936505
equal month	EVI2	interval	z log1p interval	0.000382559	0.000860865	Yes	0.969233
equal month	NDMI	antecedent	z antecedent severity	0.00958478	0.0367882	Yes	0.673913
equal month	NDMI	interval	z log1p interval	0.0326486	0.116611	No	0.817766

Table S11. Design-conditional precision and equivalence benchmark. *Column panel 4 of 4; identifier columns are repeated.*

Method	Vegetation Index	Model ID	Focal Term	Absolute Mde For 80 Percent Power	Power Status	Equivalence Margin Status
equal month	EVI2	antecedent	z antecedent severity	0.00799729	design-conditional post hoc calculation	reviewer-proposed benchmark; not a validated biological minimum
equal month	EVI2	interval	z log1p interval	0.00726225	design-conditional post hoc calculation	reviewer-proposed benchmark; not a validated biological minimum
equal month	NDMI	antecedent	z antecedent severity	0.0116545	design-conditional post hoc calculation	reviewer-proposed benchmark; not a validated biological minimum
equal month	NDMI	interval	z log1p interval	0.00976775	design-conditional post hoc calculation	reviewer-proposed benchmark; not a validated biological minimum

Table S12. Pair-within-event bootstrap model summaries. *Column panel 1 of 3; identifier columns are repeated.*

Method	Vegetation Index	Model ID	Focal Term	Observed Ols Estimate	Bootstrap Mean	Bootstrap Standard Error	Bootstrap Percentile 2 5
equal month	EVI2	antecedent	z antecedent severity	-0.00224701	-0.00148904	0.0020524	-0.00497335
equal month	EVI2	interval	z log1p interval	0.000705117	0.0012238	0.00139315	-0.00132097
equal month	NDMI	antecedent	z antecedent severity	0.00180339	0.00141574	0.00269806	-0.0045438
equal month	NDMI	interval	z log1p interval	0.00251918	0.00200939	0.00281064	-0.00360411

Table S12. Pair-within-event bootstrap model summaries. *Column panel 2 of 3; identifier columns are repeated.*

Method	Vegetation Index	Model ID	Focal Term	Bootstrap Percentile 50	Bootstrap Percentile 97 5	Probability Coefficient Above Zero	Bootstrap Replicates
equal month	EVI2	antecedent	z antecedent severity	-0.00169209	0.00262786	0.2536	10000
equal month	EVI2	interval	z log1p interval	0.00114343	0.00420795	0.8156	10000
equal month	NDMI	antecedent	z antecedent severity	0.00160183	0.00615118	0.7264	10000
equal month	NDMI	interval	z log1p interval	0.00228728	0.00674782	0.7496	10000

Table S12. Pair-within-event bootstrap model summaries. *Column panel 3 of 3; identifier columns are repeated.*

Method	Vegetation Index	Model ID	Focal Term	Random Seed	Interpretation Status
equal month	EVI2	antecedent	z antecedent severity	20261003	pair-within-event measurement bootstrap; event count unchanged
equal month	EVI2	interval	z log1p interval	20261003	pair-within-event measurement bootstrap; event count unchanged
equal month	NDMI	antecedent	z antecedent severity	20261003	pair-within-event measurement bootstrap; event count unchanged
equal month	NDMI	interval	z log1p interval	20261003	pair-within-event measurement bootstrap; event count unchanged

Table S13. Joint antecedent-severity and interval diagnostics. *Column panel 1 of 2; identifier columns are repeated.*

Method	Vegetation Index	Model ID	Term	Estimate	HC3 Se	HC3 95% CI Low	HC3 95% CI High
equal month	EVI2	joint antecedent interval	intercept	0.003355	0.0101002	-0.0213594	0.0280694
equal month	EVI2	joint antecedent interval	city Madrid	0.00161372	0.0106446	-0.0244327	0.0276601
equal month	EVI2	joint antecedent interval	city Vienna	-0.00763441	0.01395	-0.0417687	0.0264999
equal month	EVI2	joint antecedent interval	z antecedent severity	-0.00327988	0.00533639	-0.0163376	0.0097778
equal month	EVI2	joint antecedent interval	z log1p interval	0.00198141	0.00236149	-0.00379695	0.00775977
equal month	EVI2	joint antecedent interval	z current severity	-0.00385366	0.00369998	-0.0129072	0.00519985
equal month	NDMI	joint antecedent interval	intercept	-0.00102579	0.00707198	-0.0183303	0.0162787
equal month	NDMI	joint antecedent interval	city Madrid	0.0065699	0.0091618	-0.0158482	0.028988
equal month	NDMI	joint antecedent interval	city Vienna	0.000693266	0.012519	-0.0299396	0.0313261
equal month	NDMI	joint antecedent interval	z antecedent severity	0.000614932	0.00443895	-0.0102468	0.0114767
equal month	NDMI	joint antecedent interval	z log1p interval	0.00227989	0.00702509	-0.0149099	0.0194697
equal month	NDMI	joint antecedent interval	z current severity	0.00107988	0.00455871	-0.0100749	0.0122347

Table S13. Joint antecedent-severity and interval diagnostics. *Column panel 2 of 2; identifier columns are repeated.*

Method	Vegetation Index	Model ID	Term	HC3 P Two Sided	N Events	Residual df	Interpretation Status
equal month	EVI2	joint antecedent interval	intercept	0.751058	12	6	diagnostic; six residual degrees of freedom
equal month	EVI2	joint antecedent interval	city Madrid	0.884472	12	6	diagnostic; six residual degrees of freedom
equal month	EVI2	joint antecedent interval	city Vienna	0.603936	12	6	diagnostic; six residual degrees of freedom
equal month	EVI2	joint antecedent interval	z antecedent severity	0.561371	12	6	diagnostic; six residual degrees of freedom
equal month	EVI2	joint antecedent interval	z log1p interval	0.433591	12	6	diagnostic; six residual degrees of freedom
equal month	EVI2	joint antecedent interval	z current severity	0.337764	12	6	diagnostic; six residual degrees of freedom
equal month	NDMI	joint antecedent interval	intercept	0.889422	12	6	diagnostic; six residual degrees of freedom
equal month	NDMI	joint antecedent interval	city Madrid	0.500265	12	6	diagnostic; six residual degrees of freedom
equal month	NDMI	joint antecedent interval	city Vienna	0.957636	12	6	diagnostic; six residual degrees of freedom
equal month	NDMI	joint antecedent interval	z antecedent severity	0.894353	12	6	diagnostic; six residual degrees of freedom
equal month	NDMI	joint antecedent interval	z log1p interval	0.756549	12	6	diagnostic; six residual degrees of freedom
equal month	NDMI	joint antecedent interval	z current severity	0.820627	12	6	diagnostic; six residual degrees of freedom

Table S14. Event influence summary. *Column panel 1 of 2; identifier columns are repeated.*

Vegetation Index	Model ID	N Events	Max Leverage	Max Cooks Distance	N High Leverage	N High Cooks Distance	Leverage Flag Threshold
EVI2	antecedent	12	0.659578	0.741449	0	3	0.833333
EVI2	interval	12	0.695701	0.665639	0	2	0.833333
NDMI	antecedent	12	0.659578	0.375728	0	1	0.833333
NDMI	interval	12	0.695701	0.926872	0	2	0.833333

Table S14. Event influence summary. *Column panel 2 of 2; identifier columns are repeated.*

Vegetation Index	Model ID	Cooks Flag Threshold
EVI2	antecedent	0.333333
EVI2	interval	0.333333
NDMI	antecedent	0.333333
NDMI	interval	0.333333

Table S15. Reference-model selection audit. *Column panel 1 of 3; identifier columns are repeated.*

City	Vegetation Index	N Events	Selected H1 No Trend	Selected H2 No Trend	Selected H2 Trend	Selected RMSE Median	Selected RMSE Min
Berlin	EVI2	2	0	2	0	0.0451381	0.0433379
Berlin	NDMI	2	0	2	0	0.0449647	0.0426934
Madrid	EVI2	5	0	3	2	0.0399327	0.03413
Madrid	NDMI	5	0	3	2	0.0523391	0.0472716
Vienna	EVI2	5	1	4	0	0.0493972	0.0442293
Vienna	NDMI	5	0	5	0	0.0490294	0.0432231

Table S15. Reference-model selection audit. *Column panel 2 of 3; identifier columns are repeated.*

City	Vegetation Index	Selected RMSE Max	Common Heldout Pixel Days Median	Selection Tolerance	Tolerance Status	H1 No Trend RMSE Median	H2 No Trend RMSE Median
Berlin	EVI2	0.0469383	3.6574e+05	0.002	fixed numerical parsimony rule; not a biological threshold	0.0573708	0.0451381
Berlin	NDMI	0.047236	3.6574e+05	0.002	fixed numerical parsimony rule; not a biological threshold	0.0661003	0.0449647
Madrid	EVI2	0.0489084	98090	0.002	fixed numerical parsimony rule; not a biological threshold	0.0470794	0.0424224
Madrid	NDMI	0.0625709	98090	0.002	fixed numerical parsimony rule; not a biological threshold	0.0618652	0.0556856
Vienna	EVI2	0.0626869	5.9015e+05	0.002	fixed numerical parsimony rule; not a biological threshold	0.06636	0.0493972
Vienna	NDMI	0.0612518	5.9015e+05	0.002	fixed numerical parsimony rule; not a biological threshold	0.0679113	0.0490294

Table S15. Reference-model selection audit. *Column panel 3 of 3; identifier columns are repeated.*

City	Vegetation Index	H2 Trend RMSE Median
Berlin	EVI2	0.0461313
Berlin	NDMI	0.0492536
Madrid	EVI2	0.0404598
Madrid	NDMI	0.0525954
Vienna	EVI2	0.048606
Vienna	NDMI	0.0486833

Table S16. Persistent-canopy survivor-conditioning summary. *Column panel 1 of 2; identifier columns are repeated.*

City	Stratum	Matched Units	Prospective Baseline Area M2	Persistent Area M2	Baseline Overlapping Mapped Loss Area M2	Area Weighted Retained Fraction	Area Weighted Mapped Loss Fraction
Berlin	peri	30	3.1004e+06	2.8013e+06	1.9343e+05	0.903525	0.0623892
Berlin	urban	30	2.9426e+06	2.7266e+06	1.3120e+05	0.92659	0.0445856
Madrid	peri	10	6.3211e+05	3.5820e+05	2.3291e+05	0.566676	0.368471
Madrid	urban	10	6.2950e+05	3.8972e+05	2.0461e+05	0.619088	0.32503
Vienna	peri	30	4.6172e+06	4.0878e+06	4.4129e+05	0.885338	0.0955758
Vienna	urban	30	3.8908e+06	3.6324e+06	2.1710e+05	0.933569	0.0557974

Table S16. Persistent-canopy survivor-conditioning summary. *Column panel 2 of 2; identifier columns are repeated.*

City	Stratum	Interpretation Status
Berlin	peri	survivor-conditioning audit; mapped loss is not attributed to drought
Berlin	urban	survivor-conditioning audit; mapped loss is not attributed to drought
Madrid	peri	survivor-conditioning audit; mapped loss is not attributed to drought
Madrid	urban	survivor-conditioning audit; mapped loss is not attributed to drought
Vienna	peri	survivor-conditioning audit; mapped loss is not attributed to drought
Vienna	urban	survivor-conditioning audit; mapped loss is not attributed to drought

Table S17. Expanded matching and distance diagnostics. *Column panel 1 of 2; identifier columns are repeated.*

City	Variable	Stage	Urban N	Peri N	Urban Mean	Peri Mean	Urban Variance
Berlin	background mean elevation m	candidate	427	236	43.0159	44.8489	60.9202
Berlin	background mean elevation m	balanced	30	30	42.0943	41.9968	84.5265
Berlin	clim mean temperature c 1981 2010	candidate	427	236	9.6994	9.47946	0.0481059
Berlin	clim mean temperature c 1981 2010	balanced	30	30	9.50187	9.50494	0.0147736
Berlin	clim annual precipitation mm 1981 2010	candidate	427	236	578.633	579.002	145.225
Berlin	clim annual precipitation mm 1981 2010	balanced	30	30	575.357	575.318	319.751
Berlin	retained woody area ha approx	candidate	427	236	13.8244	22.6177	229.178
Berlin	retained woody area ha approx	balanced	30	30	9.08871	9.33769	86.0841
Berlin	built fraction 2020	candidate	427	236	0.190464	0.0984812	0.00470395
Berlin	built fraction 2020	balanced	30	30	0.204883	0.115982	0.00286741
Madrid	background mean elevation m	candidate	25	21	648.314	684.249	1154.7
Madrid	background mean elevation m	balanced	10	10	650.247	642.608	1076.57
Madrid	clim mean temperature c 1981 2010	candidate	25	21	14.7681	14.2404	0.0717167
Madrid	clim mean temperature c 1981 2010	balanced	10	10	14.6184	14.575	0.0295493
Madrid	clim annual precipitation mm 1981 2010	candidate	25	21	402.12	398.525	46.2695
Madrid	clim annual precipitation mm 1981 2010	balanced	10	10	401.287	400.467	22.1112
Madrid	retained woody area ha approx	candidate	25	21	4.87816	3.68847	20.2062
Madrid	retained woody area ha approx	balanced	10	10	3.89718	3.582	4.05924
Madrid	built fraction 2020	candidate	25	21	0.18101	0.0717449	0.0071321
Madrid	built fraction 2020	balanced	10	10	0.155455	0.0647654	0.00363162
Vienna	background mean elevation m	candidate	60	61	211.6	246.287	2172.85
Vienna	background mean elevation m	balanced	30	30	214.374	219.224	2712.23
Vienna	clim mean temperature c 1981 2010	candidate	60	61	10.3382	10.0297	0.111157
Vienna	clim mean temperature c 1981 2010	balanced	30	30	10.2393	10.2043	0.0876589
Vienna	clim annual precipitation mm 1981 2010	candidate	60	61	593.132	598.937	224.095
Vienna	clim annual precipitation mm 1981 2010	balanced	30	30	592.28	591.726	353.419
Vienna	retained woody area ha approx	candidate	60	61	10.7678	27.9907	127.612
Vienna	retained woody area ha approx	balanced	30	30	12.1078	13.626	112.981
Vienna	built fraction 2020	candidate	60	61	0.182419	0.093905	0.00606434
Vienna	built fraction 2020	balanced	30	30	0.176163	0.111529	0.00517651

Table S17. Expanded matching and distance diagnostics. *Column panel 2 of 2; identifier columns are repeated.*

City	Variable	Stage	Peri Variance	Urban To Peri Variance Ratio	Smd Using Fixed Candidate Pooled Sd	Design Role
Berlin	background mean elevation m	candidate	105.058	0.579871	-0.209418	matching covariate
Berlin	background mean elevation m	balanced	89.068	0.94901	0.0111349	matching covariate
Berlin	clim mean temperature c 1981 2010	candidate	0.0131821	3.64933	1.16418	matching covariate
Berlin	clim mean temperature c 1981 2010	balanced	0.0147995	0.998247	-0.016223	matching covariate
Berlin	clim annual precipitation mm 1981 2010	candidate	373.8	0.388509	-0.0245463	matching covariate
Berlin	clim annual precipitation mm 1981 2010	balanced	319.417	1.00104	0.00258406	matching covariate
Berlin	retained woody area ha approx	candidate	346.411	0.661578	-0.534296	matching covariate
Berlin	retained woody area ha approx	balanced	85.9854	1.00115	-0.0151289	matching covariate
Berlin	built fraction 2020	candidate	0.00226439	2.07736	1.48502	saved urbanization descriptor; not used for outcome-driven rematching
Berlin	built fraction 2020	balanced	0.00421932	0.67959	1.43526	saved urbanization descriptor; not used for outcome-driven rematching
Madrid	background mean elevation m	candidate	10997	0.105001	-0.478991	matching covariate
Madrid	background mean elevation m	balanced	3364.28	0.32	0.101816	matching covariate
Madrid	clim mean temperature c 1981 2010	candidate	0.476512	0.150503	1.04338	matching covariate
Madrid	clim mean temperature c 1981 2010	balanced	0.0346134	0.853698	0.0859675	matching covariate
Madrid	clim annual precipitation mm 1981 2010	candidate	408.967	0.113137	0.247385	matching covariate
Madrid	clim annual precipitation mm 1981 2010	balanced	364.176	0.0607156	0.0564335	matching covariate
Madrid	retained woody area ha approx	candidate	1.96748	10.2701	0.344643	matching covariate
Madrid	retained woody area ha approx	balanced	1.27505	3.18361	0.0913043	matching covariate
Madrid	built fraction 2020	candidate	0.000718148	9.93124	1.68266	saved urbanization descriptor; not used for outcome-driven rematching
Madrid	built fraction 2020	balanced	0.000398256	9.11879	1.3966	saved urbanization descriptor; not used for outcome-driven rematching
Vienna	background mean elevation m	candidate	4064.06	0.534651	-0.62035	matching covariate
Vienna	background mean elevation m	balanced	3232.46	0.839062	-0.0867425	matching covariate
Vienna	clim mean temperature c 1981 2010	candidate	0.136704	0.813122	0.875986	matching covariate
Vienna	clim mean temperature c 1981 2010	balanced	0.0721073	1.21567	0.0994571	matching covariate
Vienna	clim annual precipitation mm 1981 2010	candidate	734.113	0.30526	-0.264614	matching covariate
Vienna	clim annual precipitation mm 1981 2010	balanced	350.2	1.00919	0.025274	matching covariate
Vienna	retained woody area ha approx	candidate	413.77	0.308414	-1.0445	matching covariate
Vienna	retained woody area ha approx	balanced	115.087	0.981701	-0.0920667	matching covariate
Vienna	built fraction 2020	candidate	0.00155542	3.89883	1.43761	saved urbanization descriptor; not used for outcome-driven rematching
Vienna	built fraction 2020	balanced	0.00175777	2.94494	1.04975	saved urbanization descriptor; not used for outcome-driven rematching

Table S17B. Fixed matched-pair geographic distances and built-fraction descriptors. *Column panel 1 of 2; identifier columns are repeated.*

City	Pair ID	Urban Unit ID	Peri Unit ID	Centroid Distance Km	Matching Cost	Urban Built Fraction 2020	Peri Built Fraction 2020
Berlin	Berlin metric30 balanced001	Berlin 19000 2879	Berlin 18999 2873	6.49746	0.0212956	0.040973	0.077402
Berlin	Berlin metric30 balanced002	Berlin 19005 2880	Berlin 19005 2883	3.26722	0.0442006	0.202569	0.070792
Berlin	Berlin metric30 balanced003	Berlin 19007 2861	Berlin 19006 2860	1.3445	0.0203079	0.120785	0.093056
Berlin	Berlin metric30 balanced004	Berlin 19008 2851	Berlin 19047 2883	47.0712	0.0399966	0.14653	0.084568
Berlin	Berlin metric30 balanced005	Berlin 19008 2852	Berlin 19046 2885	47.1861	0.0141693	0.280349	0.062106
Berlin	Berlin metric30 balanced006	Berlin 19009 2841	Berlin 19009 2840	1.09295	0.0491855	0.196775	0.126202
Berlin	Berlin metric30 balanced007	Berlin 19009 2851	Berlin 19046 2884	46.588	0.0266926	0.251492	0.084335
Berlin	Berlin metric30 balanced008	Berlin 19009 2854	Berlin 19009 2856	2.18318	0.0399052	0.209902	0.110723
Berlin	Berlin metric30 balanced009	Berlin 19011 2842	Berlin 19012 2846	4.36095	0.0275427	0.199657	0.075373
Berlin	Berlin metric30 balanced010	Berlin 19013 2847	Berlin 19013 2845	2.18498	0.0326078	0.245414	0.235968
Berlin	Berlin metric30 balanced011	Berlin 19015 2849	Berlin 19017 2849	1.83941	0.00467058	0.203281	0.18474
Berlin	Berlin metric30 balanced012	Berlin 19017 2877	Berlin 19017 2878	1.08962	0.00878145	0.142992	0.125014
Berlin	Berlin metric30 balanced013	Berlin 19017 2886	Berlin 19011 2875	12.608	0.0353422	0.146627	0.059496
Berlin	Berlin metric30 balanced014	Berlin 19018 2877	Berlin 19016 2878	2.24712	0.0210671	0.202589	0.06628
Berlin	Berlin metric30 balanced015	Berlin 19018 2887	Berlin 19019 2886	1.50594	0.0142342	0.204492	0.202116
Berlin	Berlin metric30 balanced016	Berlin 19018 2888	Berlin 19018 2885	3.26641	0.00994522	0.221676	0.332898
Berlin	Berlin metric30 balanced017	Berlin 19019 2876	Berlin 19020 2878	2.2661	0.00648626	0.217736	0.106745
Berlin	Berlin metric30 balanced018	Berlin 19019 2878	Berlin 19020 2879	1.34351	0.00936483	0.22686	0.178292
Berlin	Berlin metric30 balanced019	Berlin 19024 2857	Berlin 19022 2854	3.56482	0.00639191	0.264118	0.057536
Berlin	Berlin metric30 balanced020	Berlin 19024 2858	Berlin 19023 2855	3.29684	0.022149	0.289284	0.056229
Berlin	Berlin metric30 balanced021	Berlin 19028 2878	Berlin 19016 2879	11.2414	0.0460254	0.289069	0.172815
Berlin	Berlin metric30 balanced022	Berlin 19031 2853	Berlin 19033 2850	3.94247	0.00431292	0.187176	0.115173
Berlin	Berlin metric30 balanced023	Berlin 19037 2848	Berlin 19037 2847	1.09272	0.0165252	0.226448	0.085541
Berlin	Berlin metric30 balanced024	Berlin 19039 2876	Berlin 19030 2882	11.15	0.025289	0.171301	0.058719
Berlin	Berlin metric30 balanced025	Berlin 19040 2875	Berlin 19009 2842	43.2617	0.0330596	0.178249	0.109223
Berlin	Berlin metric30 balanced026	Berlin 19040 2877	Berlin 19045 2883	7.54219	0.0465992	0.229697	0.100149
Berlin	Berlin metric30 balanced027	Berlin 19042 2880	Berlin 19041 2881	1.50825	0.0353535	0.252073	0.103075
Berlin	Berlin metric30 balanced028	Berlin 19044 2880	Berlin 19041 2880	2.7661	0.00417882	0.233143	0.08431
Berlin	Berlin metric30 balanced029	Berlin 19045 2884	Berlin 19029 2879	15.1158	0.0225807	0.143586	0.052744
Berlin	Berlin metric30 balanced030	Berlin 19048 2865	Berlin 19052 2866	3.71489	0.00460491	0.221637	0.20784
Madrid	Madrid metric30 balanced001	Madrid 17720 4163	Madrid 17702 4171	19.5008	3.98011	0.153457	0.055025
Madrid	Madrid metric30 balanced002	Madrid 17724 4157	Madrid 17723 4159	2.20829	0.25341	0.147851	0.044723
Madrid	Madrid metric30 balanced003	Madrid 17725 4158	Madrid 17715 4163	11.0592	0.72872	0.19252	0.057392
Madrid	Madrid metric30 balanced004	Madrid 17725 4161	Madrid 17739 4146	20.2308	0.494059	0.246803	0.04507
Madrid	Madrid metric30 balanced005	Madrid 17725 4163	Madrid 17740 4153	17.8042	0.304351	0.075984	0.075426
Madrid	Madrid metric30 balanced006	Madrid 17726 4164	Madrid 17724 4159	5.42305	0.301414	0.251687	0.051815
Madrid	Madrid metric30 balanced007	Madrid 17734 4152	Madrid 17728 4150	6.37261	0.573983	0.124775	0.073092
Madrid	Madrid metric30 balanced008	Madrid 17734 4153	Madrid 17753 4158	19.7688	6.25072	0.14145	0.093864
Madrid	Madrid metric30 balanced009	Madrid 17734 4160	Madrid 17729 4151	10.3919	0.420597	0.142105	0.100243
Madrid	Madrid metric30 balanced010	Madrid 17736 4159	Madrid 17743 4157	7.23291	1.24178	0.077914	0.051004
Vienna	Vienna metric30 balanced001	Vienna 19305 3314	Vienna 19298 3311	6.99354	0.243734	0.168797	0.099987

City	Pair ID	Urban Unit ID	Peri Unit ID	Centroid Distance Km	Matching Cost	Urban Built Fraction 2020	Peri Built Fraction 2020
Vienna	Vienna metric30 balanced002	Vienna 19305 3315	Vienna 19306 3304	11.7954	0.480197	0.179605	0.109832
Vienna	Vienna metric30 balanced003	Vienna 19306 3336	Vienna 19305 3337	1.51251	0.693683	0.18713	0.090903
Vienna	Vienna metric30 balanced004	Vienna 19307 3315	Vienna 19307 3327	12.6787	0.326488	0.175236	0.178364
Vienna	Vienna metric30 balanced005	Vienna 19307 3317	Vienna 19310 3336	19.8926	0.313827	0.268353	0.111202
Vienna	Vienna metric30 balanced006	Vienna 19307 3319	Vienna 19306 3319	0.952563	0.128879	0.152921	0.062816
Vienna	Vienna metric30 balanced007	Vienna 19307 3336	Vienna 19310 3332	5.40309	0.0351468	0.262248	0.117759
Vienna	Vienna metric30 balanced008	Vienna 19308 3312	Vienna 19308 3328	16.9065	0.382068	0.144308	0.085964
Vienna	Vienna metric30 balanced009	Vienna 19308 3313	Vienna 19308 3327	14.7931	0.17783	0.212266	0.152315
Vienna	Vienna metric30 balanced010	Vienna 19308 3318	Vienna 19310 3337	19.904	0.320592	0.249423	0.075964
Vienna	Vienna metric30 balanced011	Vienna 19309 3301	Vienna 19307 3298	3.47753	0.0792252	0.190669	0.141724
Vienna	Vienna metric30 balanced012	Vienna 19309 3304	Vienna 19313 3305	3.81276	0.218302	0.208142	0.193939
Vienna	Vienna metric30 balanced013	Vienna 19309 3311	Vienna 19308 3326	16.0055	0.282895	0.193621	0.120091
Vienna	Vienna metric30 balanced014	Vienna 19309 3312	Vienna 19307 3325	14.1167	0.271941	0.231635	0.122952
Vienna	Vienna metric30 balanced015	Vienna 19309 3318	Vienna 19320 3340	24.1901	0.67561	0.248395	0.069433
Vienna	Vienna metric30 balanced016	Vienna 19310 3305	Vienna 19311 3304	1.51408	0.254069	0.162977	0.112188
Vienna	Vienna metric30 balanced017	Vienna 19311 3310	Vienna 19311 3309	1.05751	0.715762	0.199505	0.050572
Vienna	Vienna metric30 balanced018	Vienna 19311 3312	Vienna 19307 3340	30.3137	0.032787	0.218343	0.128428
Vienna	Vienna metric30 balanced019	Vienna 19312 3310	Vienna 19309 3306	4.77925	0.236854	0.221716	0.105252
Vienna	Vienna metric30 balanced020	Vienna 19313 3309	Vienna 19312 3336	28.6681	0.37222	0.082281	0.087082
Vienna	Vienna metric30 balanced021	Vienna 19313 3310	Vienna 19314 3305	5.49679	0.120489	0.25981	0.117738
Vienna	Vienna metric30 balanced022	Vienna 19314 3307	Vienna 19315 3330	24.1997	0.126035	0.286138	0.132695
Vienna	Vienna metric30 balanced023	Vienna 19314 3309	Vienna 19310 3304	6.09149	0.536862	0.051968	0.05385
Vienna	Vienna metric30 balanced024	Vienna 19319 3317	Vienna 19316 3330	14.3966	0.378087	0.165849	0.042866
Vienna	Vienna metric30 balanced025	Vienna 19320 3316	Vienna 19328 3316	7.61895	0.685761	0.055975	0.169351
Vienna	Vienna metric30 balanced026	Vienna 19320 3317	Vienna 19326 3319	5.82218	0.319441	0.072521	0.157161
Vienna	Vienna metric30 balanced027	Vienna 19321 3317	Vienna 19326 3321	5.9305	0.436184	0.034103	0.099684
Vienna	Vienna metric30 balanced028	Vienna 19322 3317	Vienna 19324 3319	2.64967	0.028381	0.079167	0.042231
Vienna	Vienna metric30 balanced029	Vienna 19324 3320	Vienna 19325 3318	2.43215	0.200888	0.240629	0.193139
Vienna	Vienna metric30 balanced030	Vienna 19325 3321	Vienna 19325 3320	1.05686	0.0957009	0.081172	0.120402

Table S17B. Fixed matched-pair geographic distances and built-fraction descriptors. *Column panel 2 of 2; identifier columns are repeated.*

City	Pair ID	Urban Minus Peri Built Fraction 2020
Berlin	Berlin metric30 balanced001	-0.036429
Berlin	Berlin metric30 balanced002	0.131777
Berlin	Berlin metric30 balanced003	0.027729
Berlin	Berlin metric30 balanced004	0.061962
Berlin	Berlin metric30 balanced005	0.218243
Berlin	Berlin metric30 balanced006	0.070573
Berlin	Berlin metric30 balanced007	0.167157
Berlin	Berlin metric30 balanced008	0.099179
Berlin	Berlin metric30 balanced009	0.124284
Berlin	Berlin metric30 balanced010	0.00944601
Berlin	Berlin metric30 balanced011	0.018541
Berlin	Berlin metric30 balanced012	0.017978
Berlin	Berlin metric30 balanced013	0.087131
Berlin	Berlin metric30 balanced014	0.136309
Berlin	Berlin metric30 balanced015	0.00237601
Berlin	Berlin metric30 balanced016	-0.111222
Berlin	Berlin metric30 balanced017	0.110991
Berlin	Berlin metric30 balanced018	0.048568
Berlin	Berlin metric30 balanced019	0.206582
Berlin	Berlin metric30 balanced020	0.233055
Berlin	Berlin metric30 balanced021	0.116254
Berlin	Berlin metric30 balanced022	0.072003
Berlin	Berlin metric30 balanced023	0.140907
Berlin	Berlin metric30 balanced024	0.112582
Berlin	Berlin metric30 balanced025	0.069026
Berlin	Berlin metric30 balanced026	0.129548
Berlin	Berlin metric30 balanced027	0.148998
Berlin	Berlin metric30 balanced028	0.148833
Berlin	Berlin metric30 balanced029	0.090842
Berlin	Berlin metric30 balanced030	0.013797
Madrid	Madrid metric30 balanced001	0.098432
Madrid	Madrid metric30 balanced002	0.103128
Madrid	Madrid metric30 balanced003	0.135128
Madrid	Madrid metric30 balanced004	0.201733
Madrid	Madrid metric30 balanced005	0.000558004
Madrid	Madrid metric30 balanced006	0.199872
Madrid	Madrid metric30 balanced007	0.051683
Madrid	Madrid metric30 balanced008	0.047586
Madrid	Madrid metric30 balanced009	0.041862
Madrid	Madrid metric30 balanced010	0.02691
Vienna	Vienna metric30 balanced001	0.06881

City	Pair ID	Urban Minus Peri Built Fraction 2020
Vienna	Vienna metric30 balanced002	0.069773
Vienna	Vienna metric30 balanced003	0.096227
Vienna	Vienna metric30 balanced004	-0.00312799
Vienna	Vienna metric30 balanced005	0.157151
Vienna	Vienna metric30 balanced006	0.090105
Vienna	Vienna metric30 balanced007	0.144489
Vienna	Vienna metric30 balanced008	0.058344
Vienna	Vienna metric30 balanced009	0.059951
Vienna	Vienna metric30 balanced010	0.173459
Vienna	Vienna metric30 balanced011	0.048945
Vienna	Vienna metric30 balanced012	0.014203
Vienna	Vienna metric30 balanced013	0.07353
Vienna	Vienna metric30 balanced014	0.108683
Vienna	Vienna metric30 balanced015	0.178962
Vienna	Vienna metric30 balanced016	0.050789
Vienna	Vienna metric30 balanced017	0.148933
Vienna	Vienna metric30 balanced018	0.089915
Vienna	Vienna metric30 balanced019	0.116464
Vienna	Vienna metric30 balanced020	-0.004801
Vienna	Vienna metric30 balanced021	0.142072
Vienna	Vienna metric30 balanced022	0.153443
Vienna	Vienna metric30 balanced023	-0.001882
Vienna	Vienna metric30 balanced024	0.122983
Vienna	Vienna metric30 balanced025	-0.113376
Vienna	Vienna metric30 balanced026	-0.08464
Vienna	Vienna metric30 balanced027	-0.065581
Vienna	Vienna metric30 balanced028	0.036936
Vienna	Vienna metric30 balanced029	0.04749
Vienna	Vienna metric30 balanced030	-0.03923

Table S18. SPEI accumulation-scale event inventory.

City	SPEI Scale Months	Qualifying Events 1961 2024	Events Within 1985 2024 Response Record	Events Overlapping Hls 2013 2024	Annual Identifiable Antecedent Pairs
Berlin	3	31	25	10	19
Berlin	6	21	17	7	17
Berlin	12	14	10	3	10
Madrid	3	42	33	11	19
Madrid	6	32	25	8	22
Madrid	12	20	17	7	11
Vienna	3	34	24	9	18
Vienna	6	28	22	7	19
Vienna	12	26	17	8	14

Table S19. SPEI event-overlap diagnostics. *Column panel 1 of 2; identifier columns are repeated.*

City	Retained Spei6 Event ID	Comparison Scale Months	Retained Spei6 Start	Retained Spei6 End	Best Overlap Event ID	Best Overlap Start	Best Overlap End
Berlin	Berlin S06 2022-03	3	2022-03	2023-02	Berlin S03 2022-03	2022-03	2022-12
Berlin	Berlin S06 2022-03	12	2022-03	2023-02	Berlin S12 2022-05	2022-05	2023-09
Berlin	Berlin S06 2023-09	3	2023-09	2023-10	none		
Berlin	Berlin S06 2023-09	12	2023-09	2023-10	Berlin S12 2022-05	2022-05	2023-09
Vienna	Vienna S06 2017-05	3	2017-05	2017-08	Vienna S03 2017-07	2017-07	2017-08
Vienna	Vienna S06 2017-05	12	2017-05	2017-08	Vienna S12 2017-07	2017-07	2017-08
Vienna	Vienna S06 2018-08	3	2018-08	2019-04	Vienna S03 2018-09	2018-09	2018-10
Vienna	Vienna S06 2018-08	12	2018-08	2019-04	Vienna S12 2018-10	2018-10	2019-04
Vienna	Vienna S06 2020-04	3	2020-04	2020-05	Vienna S03 2020-03	2020-03	2020-05
Vienna	Vienna S06 2020-04	12	2020-04	2020-05	Vienna S12 2020-04	2020-04	2020-06
Vienna	Vienna S06 2022-02	3	2022-02	2022-12	Vienna S03 2022-02	2022-02	2022-08
Vienna	Vienna S06 2022-02	12	2022-02	2022-12	Vienna S12 2022-01	2022-01	2023-06
Vienna	Vienna S06 2024-07	3	2024-07	2024-08	none		
Vienna	Vienna S06 2024-07	12	2024-07	2024-08	none		
Madrid	Madrid S06 2016-09	3	2016-09	2017-01	Madrid S03 2016-07	2016-07	2016-10
Madrid	Madrid S06 2016-09	12	2016-09	2017-01	none		
Madrid	Madrid S06 2017-04	3	2017-04	2018-02	Madrid S03 2017-04	2017-04	2017-07
Madrid	Madrid S06 2017-04	12	2017-04	2018-02	Madrid S12 2017-03	2017-03	2018-03
Madrid	Madrid S06 2018-12	3	2018-12	2019-10	Madrid S03 2019-02	2019-02	2019-08
Madrid	Madrid S06 2018-12	12	2018-12	2019-10	Madrid S12 2019-03	2019-03	2020-04
Madrid	Madrid S06 2022-05	3	2022-05	2022-11	Madrid S03 2022-06	2022-06	2022-10
Madrid	Madrid S06 2022-05	12	2022-05	2022-11	Madrid S12 2022-06	2022-06	2023-09
Madrid	Madrid S06 2023-04	3	2023-04	2023-09	Madrid S03 2023-03	2023-03	2023-06
Madrid	Madrid S06 2023-04	12	2023-04	2023-09	Madrid S12 2022-06	2022-06	2023-09

Table S19. SPEI event-overlap diagnostics. *Column panel 2 of 2; identifier columns are repeated.*

City	Retained Spei6 Event ID	Comparison Scale Months	Overlapping Months	Month Set Jaccard	Status
Berlin	Berlin S06 2022-03	3	10	0.833333	climate-only definition comparison; vegetation outcome not re-estimated
Berlin	Berlin S06 2022-03	12	10	0.526316	climate-only definition comparison; vegetation outcome not re-estimated
Berlin	Berlin S06 2023-09	3	0	0	climate-only definition comparison; vegetation outcome not re-estimated
Berlin	Berlin S06 2023-09	12	1	0.0555556	climate-only definition comparison; vegetation outcome not re-estimated
Vienna	Vienna S06 2017-05	3	2	0.5	climate-only definition comparison; vegetation outcome not re-estimated
Vienna	Vienna S06 2017-05	12	2	0.5	climate-only definition comparison; vegetation outcome not re-estimated
Vienna	Vienna S06 2018-08	3	2	0.222222	climate-only definition comparison; vegetation outcome not re-estimated
Vienna	Vienna S06 2018-08	12	7	0.777778	climate-only definition comparison; vegetation outcome not re-estimated
Vienna	Vienna S06 2020-04	3	2	0.666667	climate-only definition comparison; vegetation outcome not re-estimated
Vienna	Vienna S06 2020-04	12	2	0.666667	climate-only definition comparison; vegetation outcome not re-estimated
Vienna	Vienna S06 2022-02	3	7	0.636364	climate-only definition comparison; vegetation outcome not re-estimated
Vienna	Vienna S06 2022-02	12	11	0.611111	climate-only definition comparison; vegetation outcome not re-estimated
Vienna	Vienna S06 2024-07	3	0	0	climate-only definition comparison; vegetation outcome not re-estimated
Vienna	Vienna S06 2024-07	12	0	0	climate-only definition comparison; vegetation outcome not re-estimated
Madrid	Madrid S06 2016-09	3	2	0.285714	climate-only definition comparison; vegetation outcome not re-estimated
Madrid	Madrid S06 2016-09	12	0	0	climate-only definition comparison; vegetation outcome not re-estimated
Madrid	Madrid S06 2017-04	3	4	0.363636	climate-only definition comparison; vegetation outcome not re-estimated
Madrid	Madrid S06 2017-04	12	11	0.846154	climate-only definition comparison; vegetation outcome not re-estimated
Madrid	Madrid S06 2018-12	3	7	0.636364	climate-only definition comparison; vegetation outcome not re-estimated
Madrid	Madrid S06 2018-12	12	8	0.470588	climate-only definition comparison; vegetation outcome not re-estimated
Madrid	Madrid S06 2022-05	3	5	0.714286	climate-only definition comparison; vegetation outcome not re-estimated
Madrid	Madrid S06 2022-05	12	6	0.352941	climate-only definition comparison; vegetation outcome not re-estimated
Madrid	Madrid S06 2023-04	3	3	0.428571	climate-only definition comparison; vegetation outcome not re-estimated
Madrid	Madrid S06 2023-04	12	6	0.375	climate-only definition comparison; vegetation outcome not re-estimated

Table S20. Descriptive residual temporal-dependence summaries.

Vegetation Index	Model ID	City	N Ordered Events	Lag1 Pearson Residual Correlation	Durbin Watson	Status
EVI2	antecedent	Berlin	2			insufficient: fewer than three events
EVI2	antecedent	Madrid	5	0.125028	1.40771	descriptive only
EVI2	antecedent	Vienna	5	0.404263	1.13094	descriptive only
EVI2	interval	Berlin	2			insufficient: fewer than three events
EVI2	interval	Madrid	5	-0.000221939	1.54911	descriptive only
EVI2	interval	Vienna	5	0.026211	1.90635	descriptive only
NDMI	antecedent	Berlin	2			insufficient: fewer than three events
NDMI	antecedent	Madrid	5	0.586901	0.815486	descriptive only
NDMI	antecedent	Vienna	5	0.182672	1.32745	descriptive only
NDMI	interval	Berlin	2			insufficient: fewer than three events
NDMI	interval	Madrid	5	0.569627	0.828868	descriptive only
NDMI	interval	Vienna	5	0.353456	1.09654	descriptive only

Table S21. Thirty-metre inner-buffer event outcomes. *Column panel 1 of 2; identifier columns are repeated.*

City	Event ID	Fixed Candidate Pairs	Supported Pairs	Required Supported Pairs	Supported Pair Fraction	Inner30 Event Gate Pass	Median Equal Month Anomaly EVI2 Urban Minus Peri
Berlin	Berlin S06 2022-03	30	27	24	0.9	Yes	0.00201852
Berlin	Berlin S06 2023-09	30	27	24	0.9	Yes	-0.00320259
Madrid	Madrid S06 2016-09	10	10	8	1	Yes	0.00697246
Madrid	Madrid S06 2017-04	10	10	8	1	Yes	-0.00145709
Madrid	Madrid S06 2018-12	9	9	8	1	Yes	-0.00986559
Madrid	Madrid S06 2022-05	10	10	8	1	Yes	0.00608887
Madrid	Madrid S06 2023-04	10	10	8	1	Yes	0.0116197
Vienna	Vienna S06 2017-05	30	30	24	1	Yes	0.000679343
Vienna	Vienna S06 2018-08	30	30	24	1	Yes	-0.00315978
Vienna	Vienna S06 2020-04	30	30	24	1	Yes	0.00394089
Vienna	Vienna S06 2022-02	30	30	24	1	Yes	-0.00017514
Vienna	Vienna S06 2024-07	30	30	24	1	Yes	0.00141704

Table S21. Thirty-metre inner-buffer event outcomes. *Column panel 2 of 2; identifier columns are repeated.*

City	Event ID	Median Equal Month Anomaly NDMI Urban Minus Peri	Median Equal Valid Day Anomaly EVI2 Urban Minus Peri	Median Equal Valid Day Anomaly NDMI Urban Minus Peri	Median Acquisition Weighted Anomaly EVI2 Urban Minus Peri	Median Acquisition Weighted Anomaly NDMI Urban Minus Peri
Berlin	Berlin S06 2022-03	0.000322015	0.00180164	0.000337037	0.00255636	-0.00148531
Berlin	Berlin S06 2023-09	-0.00366097	-0.00274096	-0.00278255	-0.00299091	-0.00329343
Madrid	Madrid S06 2016-09	-0.0115188	0.00881697	-0.0135655	0.00839783	-0.0136888
Madrid	Madrid S06 2017-04	0.00091771	-0.00513753	-0.00188185	-0.00549638	-0.00241697
Madrid	Madrid S06 2018-12	0.000601772	-0.0117818	-0.000182906	-0.0126306	-0.000170927
Madrid	Madrid S06 2022-05	0.0165551	0.00636919	0.0140782	0.00635977	0.0143115
Madrid	Madrid S06 2023-04	0.0181033	0.0124088	0.0177638	0.0119488	0.0176497
Vienna	Vienna S06 2017-05	-0.00573472	-0.00142758	-0.0030561	-0.0012319	-0.00409979
Vienna	Vienna S06 2018-08	-0.00197731	-0.00268083	-0.00150075	-0.00308732	-0.00143516
Vienna	Vienna S06 2020-04	0.000983381	0.0036872	0.000212775	0.00328467	0.00119069
Vienna	Vienna S06 2022-02	0.00357713	-0.000438559	0.00201926	-0.00120831	0.00159006
Vienna	Vienna S06 2024-07	0.00212095	0.00108399	0.00197853	0.00118661	0.00222735

Table S22. Thirty-metre inner-buffer model coefficients. *Column panel 1 of 3; identifier columns are repeated.*

Vegetation Index	Model ID	Term	Method	Inner30 Estimate	Inner30 HC3 Se	Inner30 HC3 95% CI Low	Inner30 HC3 95% CI High
EVI2	antecedent	intercept	equal month	0.00245959	0.0107575	-0.0229778	0.0278969
EVI2	antecedent	city Madrid	equal month	0.00112058	0.0118483	-0.0268963	0.0291375
EVI2	antecedent	city Vienna	equal month	-0.00404827	0.0127276	-0.0341443	0.0260478
EVI2	antecedent	z antecedent severity	equal month	-0.00249111	0.00426801	-0.0125833	0.00760113
EVI2	antecedent	z current severity	equal month	-0.00297021	0.00378831	-0.0119281	0.00598771
EVI2	interval	intercept	equal month	0.000115693	0.01029	-0.0242163	0.0244477
EVI2	interval	city Madrid	equal month	0.00326445	0.0111291	-0.0230517	0.0295806
EVI2	interval	city Vienna	equal month	-0.000566789	0.0105243	-0.0254529	0.0243193
EVI2	interval	z log1p interval	equal month	0.000722838	0.00223157	-0.00455398	0.00599965
EVI2	interval	z current severity	equal month	-0.00182642	0.0028709	-0.00861502	0.00496218
NDMI	antecedent	intercept	equal month	-0.00427943	0.00492289	-0.0159202	0.00736136
NDMI	antecedent	city Madrid	equal month	0.008437	0.00798034	-0.0104335	0.0273075
NDMI	antecedent	city Vienna	equal month	0.00589154	0.00817231	-0.0134329	0.025216
NDMI	antecedent	z antecedent severity	equal month	0.00213716	0.00433584	-0.00811548	0.0123898
NDMI	antecedent	z current severity	equal month	0.00252215	0.00422099	-0.0074589	0.0125032
NDMI	interval	intercept	equal month	-0.00161701	0.00302713	-0.00877505	0.00554102
NDMI	interval	city Madrid	equal month	0.00748536	0.00869097	-0.0130655	0.0280362
NDMI	interval	city Vienna	equal month	0.000453384	0.00572417	-0.0130821	0.0139889
NDMI	interval	z log1p interval	equal month	0.00255661	0.00586777	-0.0113185	0.0164317
NDMI	interval	z current severity	equal month	0.00143972	0.00262821	-0.00477501	0.00765445

Table S22. Thirty-metre inner-buffer model coefficients. *Column panel 2 of 3; identifier columns are repeated.*

Vegetation Index	Model ID	Term	Inner30 HC3 P	N Events	Residual df	Primary Estimate	Inner30 Minus Primary Estimate
EVI2	antecedent	intercept	0.825684	12	7		
EVI2	antecedent	city Madrid	0.927301	12	7		
EVI2	antecedent	city Vienna	0.759711	12	7		
EVI2	antecedent	z antecedent severity	0.577756	12	7	-0.00224701	-0.000244096
EVI2	antecedent	z current severity	0.458717	12	7		
EVI2	interval	intercept	0.991343	12	7		
EVI2	interval	city Madrid	0.777779	12	7		
EVI2	interval	city Vienna	0.958555	12	7		
EVI2	interval	z log1p interval	0.755465	12	7	0.000705117	1.7721e-05
EVI2	interval	z current severity	0.544888	12	7		
NDMI	antecedent	intercept	0.413499	12	7		
NDMI	antecedent	city Madrid	0.325524	12	7		
NDMI	antecedent	city Vienna	0.494328	12	7		
NDMI	antecedent	z antecedent severity	0.637164	12	7	0.00180339	0.000333771
NDMI	antecedent	z current severity	0.568973	12	7		
NDMI	interval	intercept	0.609759	12	7		
NDMI	interval	city Madrid	0.417607	12	7		
NDMI	interval	city Vienna	0.939086	12	7		
NDMI	interval	z log1p interval	0.676171	12	7	0.00251918	3.7437e-05
NDMI	interval	z current severity	0.600856	12	7		

Table S22. Thirty-metre inner-buffer model coefficients. *Column panel 3 of 3; identifier columns are repeated.*

Vegetation Index	Model ID	Term	Focal Sign Changed From Primary
EVI2	antecedent	intercept	
EVI2	antecedent	city Madrid	
EVI2	antecedent	city Vienna	
EVI2	antecedent	z antecedent severity	No
EVI2	antecedent	z current severity	
EVI2	interval	intercept	
EVI2	interval	city Madrid	
EVI2	interval	city Vienna	
EVI2	interval	z log1p interval	No
EVI2	interval	z current severity	
NDMI	antecedent	intercept	
NDMI	antecedent	city Madrid	
NDMI	antecedent	city Vienna	
NDMI	antecedent	z antecedent severity	No
NDMI	antecedent	z current severity	
NDMI	interval	intercept	
NDMI	interval	city Madrid	
NDMI	interval	city Vienna	
NDMI	interval	z log1p interval	No
NDMI	interval	z current severity	

Table S23. Full-climate antecedent audit. *Column panel 1 of 3; identifier columns are repeated.*

City	Current Event ID	Current Start	Current End	Full Climate Antecedent Event ID	Antecedent Start	Antecedent End	Antecedent Severity
Berlin	Berlin S06 2014-03	2014-03	2014-06	Berlin S06 2012-03	2012-03	2012-04	0.519792
Berlin	Berlin S06 2015-06	2015-06	2015-10	Berlin S06 2014-03	2014-03	2014-06	2.32303
Berlin	Berlin S06 2016-05	2016-05	2017-02	Berlin S06 2015-06	2015-06	2015-10	2.13105
Berlin	Berlin S06 2018-05	2018-05	2020-01	Berlin S06 2016-05	2016-05	2017-02	3.29158
Berlin	Berlin S06 2020-04	2020-04	2021-01	Berlin S06 2018-05	2018-05	2020-01	19.5452
Berlin	Berlin S06 2022-03	2022-03	2023-02	Berlin S06 2020-04	2020-04	2021-01	5.0998
Berlin	Berlin S06 2023-09	2023-09	2023-10	Berlin S06 2022-03	2022-03	2023-02	9.31831
Hamburg	Hamburg S06 2014-03	2014-03	2014-04	Hamburg S06 2011-04	2011-04	2011-06	1.94653
Hamburg	Hamburg S06 2016-09	2016-09	2017-02	Hamburg S06 2014-03	2014-03	2014-04	0.619464
Helsinki	Helsinki S06 2018-06	2018-06	2018-12	Helsinki S06 2013-06	2013-06	2013-09	0.550167
Helsinki	Helsinki S06 2019-08	2019-08	2019-09	Helsinki S06 2018-06	2018-06	2018-12	4.96645
Helsinki	Helsinki S06 2022-08	2022-08	2022-11	Helsinki S06 2019-08	2019-08	2019-09	0.257549
Madrid	Madrid S06 2014-07	2014-07	2014-10	Madrid S06 2013-09	2013-09	2013-12	1.13066
Madrid	Madrid S06 2015-05	2015-05	2016-01	Madrid S06 2014-07	2014-07	2014-10	1.36652
Madrid	Madrid S06 2016-09	2016-09	2017-01	Madrid S06 2015-05	2015-05	2016-01	4.88409
Madrid	Madrid S06 2017-04	2017-04	2018-02	Madrid S06 2016-09	2016-09	2017-01	1.04782
Madrid	Madrid S06 2018-12	2018-12	2019-10	Madrid S06 2017-04	2017-04	2018-02	8.09824
Madrid	Madrid S06 2022-05	2022-05	2022-11	Madrid S06 2018-12	2018-12	2019-10	6.61616
Madrid	Madrid S06 2023-04	2023-04	2023-09	Madrid S06 2022-05	2022-05	2022-11	3.89109
Vienna	Vienna S06 2014-03	2014-03	2014-04	Vienna S06 2011-03	2011-03	2012-09	10.7495
Vienna	Vienna S06 2015-07	2015-07	2015-09	Vienna S06 2014-03	2014-03	2014-04	0.749982
Vienna	Vienna S06 2017-05	2017-05	2017-08	Vienna S06 2015-07	2015-07	2015-09	1.42353
Vienna	Vienna S06 2018-08	2018-08	2019-04	Vienna S06 2017-05	2017-05	2017-08	0.60058
Vienna	Vienna S06 2020-04	2020-04	2020-05	Vienna S06 2018-08	2018-08	2019-04	3.53089
Vienna	Vienna S06 2022-02	2022-02	2022-12	Vienna S06 2020-04	2020-04	2020-05	0.826381
Vienna	Vienna S06 2024-07	2024-07	2024-08	Vienna S06 2022-02	2022-02	2022-12	8.69813

Table S23. Full-climate antecedent audit. *Column panel 2 of 3; identifier columns are repeated.*

City	Current Event ID	Drought Free Interval Months	Fixed Pairs	Supported Pairs	Supported Pair Fraction	Support Gate Pass	V7 Antecedent Within Hls Record
Berlin	Berlin S06 2014-03	22	30	0	0	No	No
Berlin	Berlin S06 2015-06	11	30	0	0	No	Yes
Berlin	Berlin S06 2016-05	6	30	0	0	No	Yes
Berlin	Berlin S06 2018-05	14	30	6	0.2	No	Yes
Berlin	Berlin S06 2020-04	2	30	4	0.133333	No	Yes
Berlin	Berlin S06 2022-03	13	30	30	1	Yes	Yes
Berlin	Berlin S06 2023-09	6	30	30	1	Yes	Yes
Hamburg	Hamburg S06 2014-03	32	30	10	0.333333	No	No
Hamburg	Hamburg S06 2016-09	28	30	9	0.3	No	Yes
Helsinki	Helsinki S06 2018-06	56	30	20	0.666667	No	No
Helsinki	Helsinki S06 2019-08	7	30	23	0.766667	No	Yes
Helsinki	Helsinki S06 2022-08	34	30	23	0.766667	No	Yes
Madrid	Madrid S06 2014-07	6	10	0	0	No	No
Madrid	Madrid S06 2015-05	6	10	0	0	No	Yes
Madrid	Madrid S06 2016-09	7	10	10	1	Yes	Yes
Madrid	Madrid S06 2017-04	2	10	10	1	Yes	Yes
Madrid	Madrid S06 2018-12	9	10	9	0.9	Yes	Yes
Madrid	Madrid S06 2022-05	30	10	10	1	Yes	Yes
Madrid	Madrid S06 2023-04	4	10	10	1	Yes	Yes
Vienna	Vienna S06 2014-03	17	30	4	0.133333	No	No
Vienna	Vienna S06 2015-07	14	30	5	0.166667	No	Yes
Vienna	Vienna S06 2017-05	19	30	30	1	Yes	Yes
Vienna	Vienna S06 2018-08	11	30	30	1	Yes	Yes
Vienna	Vienna S06 2020-04	11	30	30	1	Yes	Yes
Vienna	Vienna S06 2022-02	20	30	30	1	Yes	Yes
Vienna	Vienna S06 2024-07	18	30	30	1	Yes	Yes

Table S23. Full-climate antecedent audit. *Column panel 3 of 3; identifier columns are repeated.*

City	Current Event ID	V7 Status	V9 Status	Status Changed
Berlin	Berlin S06 2014-03	Antecedent history outside HLS record	Affected-month support below 80% of fixed pairs	Yes
Berlin	Berlin S06 2015-06	Affected-month support below 80% of fixed pairs	Affected-month support below 80% of fixed pairs	No
Berlin	Berlin S06 2016-05	Affected-month support below 80% of fixed pairs	Affected-month support below 80% of fixed pairs	No
Berlin	Berlin S06 2018-05	Affected-month support below 80% of fixed pairs	Affected-month support below 80% of fixed pairs	No
Berlin	Berlin S06 2020-04	Affected-month support below 80% of fixed pairs	Affected-month support below 80% of fixed pairs	No
Berlin	Berlin S06 2022-03	Retained analytical event	Retained analytical event	No
Berlin	Berlin S06 2023-09	Retained analytical event	Retained analytical event	No
Hamburg	Hamburg S06 2014-03	Antecedent history outside HLS record	Affected-month support below 80% of fixed pairs	Yes
Hamburg	Hamburg S06 2016-09	Affected-month support below 80% of fixed pairs	Affected-month support below 80% of fixed pairs	No
Helsinki	Helsinki S06 2018-06	Antecedent history outside HLS record	Affected-month support below 80% of fixed pairs	Yes
Helsinki	Helsinki S06 2019-08	Affected-month support below 80% of fixed pairs	Affected-month support below 80% of fixed pairs	No
Helsinki	Helsinki S06 2022-08	Affected-month support below 80% of fixed pairs	Affected-month support below 80% of fixed pairs	No
Madrid	Madrid S06 2014-07	Antecedent history outside HLS record	Affected-month support below 80% of fixed pairs	Yes
Madrid	Madrid S06 2015-05	Affected-month support below 80% of fixed pairs	Affected-month support below 80% of fixed pairs	No
Madrid	Madrid S06 2016-09	Retained analytical event	Retained analytical event	No
Madrid	Madrid S06 2017-04	Retained analytical event	Retained analytical event	No
Madrid	Madrid S06 2018-12	Retained analytical event	Retained analytical event	No
Madrid	Madrid S06 2022-05	Retained analytical event	Retained analytical event	No
Madrid	Madrid S06 2023-04	Retained analytical event	Retained analytical event	No
Vienna	Vienna S06 2014-03	Antecedent history outside HLS record	Affected-month support below 80% of fixed pairs	Yes
Vienna	Vienna S06 2015-07	Affected-month support below 80% of fixed pairs	Affected-month support below 80% of fixed pairs	No
Vienna	Vienna S06 2017-05	Retained analytical event	Retained analytical event	No
Vienna	Vienna S06 2018-08	Retained analytical event	Retained analytical event	No
Vienna	Vienna S06 2020-04	Retained analytical event	Retained analytical event	No
Vienna	Vienna S06 2022-02	Retained analytical event	Retained analytical event	No
Vienna	Vienna S06 2024-07	Retained analytical event	Retained analytical event	No

Table S24. Growing-season-aligned event predictors. *Column panel 1 of 6; identifier columns are repeated.*

City	Event ID	Antecedent Event ID	Antecedent Severity	Current Severity	Duration Months	Drought Free Interval Months	Log1p Interval
Berlin	Berlin S06 2022-03	Berlin S06 2020-04	5.0998	9.31831	12	13	2.63906
Berlin	Berlin S06 2023-09	Berlin S06 2022-03	9.31831	0.628563	2	6	1.94591
Madrid	Madrid S06 2016-09	Madrid S06 2015-05	4.88409	1.04782	5	7	2.07944
Madrid	Madrid S06 2017-04	Madrid S06 2016-09	1.04782	8.09824	11	2	1.09861
Madrid	Madrid S06 2018-12	Madrid S06 2017-04	8.09824	6.61616	11	9	2.30259
Madrid	Madrid S06 2022-05	Madrid S06 2018-12	6.61616	3.89109	7	30	3.43399
Madrid	Madrid S06 2023-04	Madrid S06 2022-05	3.89109	3.29826	6	4	1.60944
Vienna	Vienna S06 2017-05	Vienna S06 2015-07	1.42353	0.60058	4	19	2.99573
Vienna	Vienna S06 2018-08	Vienna S06 2017-05	0.60058	3.53089	9	11	2.48491
Vienna	Vienna S06 2020-04	Vienna S06 2018-08	3.53089	0.826381	2	11	2.48491
Vienna	Vienna S06 2022-02	Vienna S06 2020-04	0.826381	8.69813	11	20	3.04452
Vienna	Vienna S06 2024-07	Vienna S06 2022-02	8.69813	0.343773	2	18	2.94444

Table S24. Growing-season-aligned event predictors. *Column panel 2 of 6; identifier columns are repeated.*

City	Event ID	Z Antecedent Severity	Z Current Severity	Z Log1p Interval	Z Duration Months	Median Equal Month Anomaly EVI2 Urban Minus Peri	Median Equal Month Anomaly NDMI Urban Minus Peri
Berlin	Berlin S06 2022-03	0.188609	1.57557	0.327329	1.33134	0.000843794	-0.000112929
Berlin	Berlin S06 2023-09	1.52161	-0.95511	-0.717773	-1.24545	-0.00290778	-0.00110712
Madrid	Madrid S06 2016-09	0.120447	-0.833013	-0.516439	-0.472411	0.00873104	-0.0105392
Madrid	Madrid S06 2017-04	-1.09177	1.22025	-1.9953	1.07366	-0.00181707	0.000706742
Madrid	Madrid S06 2018-12	1.13608	0.788633	-0.179991	1.07366	-0.00954836	0.000601772
Madrid	Madrid S06 2022-05	0.667761	-0.00497764	1.5259	0.0429465	0.00549088	0.0163639
Madrid	Madrid S06 2023-04	-0.193331	-0.177627	-1.22509	-0.214732	0.0113255	0.016602
Vienna	Vienna S06 2017-05	-0.973052	-0.96326	0.865111	-0.73009	9.8546e-05	-0.00347139
Vienna	Vienna S06 2018-08	-1.2331	-0.109877	0.0949063	0.558304	-0.00557183	-0.00163757
Vienna	Vienna S06 2020-04	-0.30715	-0.897501	0.0949063	-1.24545	0.00328693	0.00161778
Vienna	Vienna S06 2022-02	-1.16175	1.39496	0.938675	1.07366	-0.000951718	0.00316973
Vienna	Vienna S06 2024-07	1.32564	-1.03805	0.787772	-1.24545	0.00117658	0.0018127

Table S24. Growing-season-aligned event predictors. *Column panel 3 of 6; identifier columns are repeated.*

City	Event ID	Median Equal Valid Day Anomaly EVI2 Urban Minus Peri	Median Equal Valid Day Anomaly NDMI Urban Minus Peri	Median Acquisition Weighted Anomaly EVI2 Urban Minus Peri	Median Acquisition Weighted Anomaly NDMI Urban Minus Peri	Fixed Candidate Pairs	Supported Pairs
Berlin	Berlin S06 2022-03	0.00106203	0.000667531	0.00243526	-7.3434e-05	30	27
Berlin	Berlin S06 2023-09	-0.00237856	-0.0016944	-0.00306274	-0.00349059	30	27
Madrid	Madrid S06 2016-09	0.00942172	-0.0136964	0.00896977	-0.0135617	10	10
Madrid	Madrid S06 2017-04	-0.00494253	-0.00183297	-0.00525198	-0.00229268	10	10
Madrid	Madrid S06 2018-12	-0.0112588	-0.000182906	-0.0120655	-0.000170927	9	9
Madrid	Madrid S06 2022-05	0.0051508	0.0143092	0.00566737	0.0145448	10	10
Madrid	Madrid S06 2023-04	0.0125139	0.0163562	0.0120267	0.0162809	10	10
Vienna	Vienna S06 2017-05	-0.000874895	-0.00135232	-0.000650593	-0.00212593	30	30
Vienna	Vienna S06 2018-08	-0.00511298	-0.00140626	-0.00517505	-0.00111423	30	30
Vienna	Vienna S06 2020-04	0.00339416	0.000530443	0.00306188	0.000990974	30	30
Vienna	Vienna S06 2022-02	-0.000763477	0.00121099	-0.000737995	0.000189771	30	30
Vienna	Vienna S06 2024-07	0.00136937	0.00161633	0.00164635	0.0016563	30	30

Table S24. Growing-season-aligned event predictors. *Column panel 4 of 6; identifier columns are repeated.*

City	Event ID	Supported Pair Fraction	City Growing Season	Antecedent Start	Antecedent End	Antecedent Full Severity Reconstructed	Antecedent Growing Season Aligned Severity
Berlin	Berlin S06 2022-03	0.9	04-10	2020-04	2021-01	5.0998	4.14436
Berlin	Berlin S06 2023-09	0.9	04-10	2022-03	2023-02	9.31831	6.87128
Madrid	Madrid S06 2016-09	1	03-10	2015-05	2016-01	4.88409	3.53791
Madrid	Madrid S06 2017-04	1	03-10	2016-09	2017-01	1.04782	0.646584
Madrid	Madrid S06 2018-12	1	03-10	2017-04	2018-02	8.09824	5.40233
Madrid	Madrid S06 2022-05	1	03-10	2018-12	2019-10	6.61616	5.99627
Madrid	Madrid S06 2023-04	1	03-10	2022-05	2022-11	3.89109	3.21034
Vienna	Vienna S06 2017-05	1	04-10	2015-07	2015-09	1.42353	1.42353
Vienna	Vienna S06 2018-08	1	04-10	2017-05	2017-08	0.60058	0.60058
Vienna	Vienna S06 2020-04	1	04-10	2018-08	2019-04	3.53089	1.60432
Vienna	Vienna S06 2022-02	1	04-10	2020-04	2020-05	0.826381	0.826381
Vienna	Vienna S06 2024-07	1	04-10	2022-02	2022-12	8.69813	5.95444

Table S24. Growing-season-aligned event predictors. *Column panel 5 of 6; identifier columns are repeated.*

City	Event ID	Antecedent Dormant Season Severity	Antecedent Event Months	Antecedent Growing Season Event Months	Current Start	Current End	Current Full Severity Reconstructed
Berlin	Berlin S06 2022-03	0.955441	10	7	2022-03	2023-02	9.31831
Berlin	Berlin S06 2023-09	2.44704	12	7	2023-09	2023-10	0.628563
Madrid	Madrid S06 2016-09	1.34618	9	6	2016-09	2017-01	1.04782
Madrid	Madrid S06 2017-04	0.401233	5	2	2017-04	2018-02	8.09824
Madrid	Madrid S06 2018-12	2.69591	11	7	2018-12	2019-10	6.61616
Madrid	Madrid S06 2022-05	0.619891	11	8	2022-05	2022-11	3.89109
Madrid	Madrid S06 2023-04	0.680747	7	6	2023-04	2023-09	3.29826
Vienna	Vienna S06 2017-05	0	3	3	2017-05	2017-08	0.60058
Vienna	Vienna S06 2018-08	0	4	4	2018-08	2019-04	3.53089
Vienna	Vienna S06 2020-04	1.92657	9	4	2020-04	2020-05	0.826381
Vienna	Vienna S06 2022-02	0	2	2	2022-02	2022-12	8.69813
Vienna	Vienna S06 2024-07	2.74369	11	7	2024-07	2024-08	0.343773

Table S24. Growing-season-aligned event predictors. *Column panel 6 of 6; identifier columns are repeated.*

City	Event ID	Current Growing Season Aligned Severity	Current Dormant Season Severity	Current Event Months	Current Growing Season Event Months	Z Antecedent Growing Season Aligned Severity	Z Current Growing Season Aligned Severity
Berlin	Berlin S06 2022-03	6.87128	2.44704	12	7	0.34211	1.56381
Berlin	Berlin S06 2023-09	0.628563	0	2	2	1.51878	-0.9249
Madrid	Madrid S06 2016-09	0.646584	0.401233	5	2	0.0804241	-0.917716
Madrid	Madrid S06 2017-04	5.40233	2.69591	11	7	-1.16719	0.978204
Madrid	Madrid S06 2018-12	5.99627	0.619891	11	8	0.884925	1.21498
Madrid	Madrid S06 2022-05	3.21034	0.680747	7	6	1.14121	0.104349
Madrid	Madrid S06 2023-04	3.29826	0	6	6	-0.060921	0.139396
Vienna	Vienna S06 2017-05	0.60058	0	4	4	-0.831932	-0.936056
Vienna	Vienna S06 2018-08	1.60432	1.92657	9	4	-1.18704	-0.535907
Vienna	Vienna S06 2020-04	0.826381	0	2	2	-0.753924	-0.846038
Vienna	Vienna S06 2022-02	5.95444	2.74369	11	7	-1.08961	1.19831
Vienna	Vienna S06 2024-07	0.343773	0	2	2	1.12316	-1.03843

Table S25. Growing-season-aligned model results. *Column panel 1 of 3; identifier columns are repeated.*

Vegetation Index	Model ID	Term	Method	Estimate	HC3 Se	HC3 95% CI Low	HC3 95% CI High
EVI2	antecedent	intercept	growing season aligned severity	0.000899367	0.0106051	-0.0241777	0.0259765
EVI2	antecedent	city Madrid	growing season aligned severity	0.00296964	0.0115129	-0.0242539	0.0301932
EVI2	antecedent	city Vienna	growing season aligned severity	-0.00309682	0.0122422	-0.032045	0.0258514
EVI2	antecedent	z antecedent growing season aligned severity	growing season aligned severity	-0.00113405	0.00342111	-0.00922368	0.00695559
EVI2	antecedent	z current growing season aligned severity	growing season aligned severity	-0.00274276	0.00383212	-0.0118043	0.00631877
EVI2	interval	intercept	growing season aligned severity	-8.7903e-05	0.00997456	-0.023674	0.0234982
EVI2	interval	city Madrid	growing season aligned severity	0.00407861	0.0106803	-0.0211763	0.0293335
EVI2	interval	city Vienna	growing season aligned severity	-0.00183634	0.0104244	-0.0264862	0.0228135
EVI2	interval	z log1p interval	growing season aligned severity	0.000876423	0.00177513	-0.0033211	0.00507395
EVI2	interval	z current growing season aligned severity	growing season aligned severity	-0.00241972	0.00315557	-0.00988146	0.00504202
NDMI	antecedent	intercept	growing season aligned severity	-0.00435229	0.00517412	-0.0165871	0.00788256
NDMI	antecedent	city Madrid	growing season aligned severity	0.0077472	0.00801132	-0.0111966	0.026691
NDMI	antecedent	city Vienna	growing season aligned severity	0.00749958	0.00891045	-0.0135703	0.0285694
NDMI	antecedent	z antecedent growing season aligned severity	growing season aligned severity	0.00311196	0.00389267	-0.00609274	0.0123167
NDMI	antecedent	z current growing season aligned severity	growing season aligned severity	0.00265067	0.00411173	-0.00707202	0.0123734
NDMI	interval	intercept	growing season aligned severity	-0.00055858	0.00567846	-0.013986	0.0128688
NDMI	interval	city Madrid	growing season aligned severity	0.00605914	0.0101583	-0.0179615	0.0300798
NDMI	interval	city Vienna	growing season aligned severity	8.2733e-05	0.00807541	-0.0190126	0.019178
NDMI	interval	z log1p interval	growing season aligned severity	0.00240883	0.00626001	-0.0123937	0.0172114
NDMI	interval	z current growing season aligned severity	growing season aligned severity	0.00131102	0.00340864	-0.00674913	0.00937118

Table S25. Growing-season-aligned model results. *Column panel 2 of 3; identifier columns are repeated.*

Vegetation Index	Model ID	Term	HC3 P Two Sided	N Events	Residual df	Primary V8 Focal Estimate	Focal Direction Preserved
EVI2	antecedent	intercept	0.934791	12	7		
EVI2	antecedent	city Madrid	0.803871	12	7		
EVI2	antecedent	city Vienna	0.807565	12	7		
EVI2	antecedent	z antecedent growing season aligned severity	0.749981	12	7	-0.00224701	Yes
EVI2	antecedent	z current growing season aligned severity	0.497333	12	7		
EVI2	interval	intercept	0.993214	12	7		
EVI2	interval	city Madrid	0.713878	12	7		
EVI2	interval	city Vienna	0.865158	12	7		
EVI2	interval	z log1p interval	0.636615	12	7	0.000705117	Yes
EVI2	interval	z current growing season aligned severity	0.468263	12	7		
NDMI	antecedent	intercept	0.428051	12	7		
NDMI	antecedent	city Madrid	0.365742	12	7		
NDMI	antecedent	city Vienna	0.427792	12	7		
NDMI	antecedent	z antecedent growing season aligned severity	0.450306	12	7	0.00180339	Yes
NDMI	antecedent	z current growing season aligned severity	0.539691	12	7		
NDMI	interval	intercept	0.924397	12	7		
NDMI	interval	city Madrid	0.569641	12	7		
NDMI	interval	city Vienna	0.992112	12	7		
NDMI	interval	z log1p interval	0.711812	12	7	0.00251918	Yes
NDMI	interval	z current growing season aligned severity	0.711939	12	7		

Table S25. Growing-season-aligned model results. *Column panel 3 of 3; identifier columns are repeated.*

Vegetation Index	Model ID	Term	Interpretation Status
EVI2	antecedent	intercept	post hoc response-window-alignment sensitivity
EVI2	antecedent	city Madrid	post hoc response-window-alignment sensitivity
EVI2	antecedent	city Vienna	post hoc response-window-alignment sensitivity
EVI2	antecedent	z antecedent growing season aligned severity	post hoc response-window-alignment sensitivity
EVI2	antecedent	z current growing season aligned severity	post hoc response-window-alignment sensitivity
EVI2	interval	intercept	post hoc response-window-alignment sensitivity
EVI2	interval	city Madrid	post hoc response-window-alignment sensitivity
EVI2	interval	city Vienna	post hoc response-window-alignment sensitivity
EVI2	interval	z log1p interval	post hoc response-window-alignment sensitivity
EVI2	interval	z current growing season aligned severity	post hoc response-window-alignment sensitivity
NDMI	antecedent	intercept	post hoc response-window-alignment sensitivity
NDMI	antecedent	city Madrid	post hoc response-window-alignment sensitivity
NDMI	antecedent	city Vienna	post hoc response-window-alignment sensitivity
NDMI	antecedent	z antecedent growing season aligned severity	post hoc response-window-alignment sensitivity
NDMI	antecedent	z current growing season aligned severity	post hoc response-window-alignment sensitivity
NDMI	interval	intercept	post hoc response-window-alignment sensitivity
NDMI	interval	city Madrid	post hoc response-window-alignment sensitivity
NDMI	interval	city Vienna	post hoc response-window-alignment sensitivity
NDMI	interval	z log1p interval	post hoc response-window-alignment sensitivity
NDMI	interval	z current growing season aligned severity	post hoc response-window-alignment sensitivity

Table S26A. Annual HLS source-inventory availability across all 140 fixed analytical units before pixel-level quality control. *Column panel 1 of 2; identifier columns are repeated.*

Geography	Year	Unit Year Records	Inventory Unit Days	L30 Only Unit Days	S30 Only Unit Days	Both Sensor Unit Days	L30 Acquisition Records
All cities	2013	140	2673	2673	0	0	4456
All cities	2014	140	2667	2667	0	0	4526
All cities	2015	140	3063	3063	0	0	5184
All cities	2016	140	7431	2474	4498	459	4928
All cities	2017	140	8895	2208	6058	629	4719
All cities	2018	140	12994	1873	9853	1268	5368
All cities	2019	140	11727	1842	8648	1237	5164
All cities	2020	140	11985	1833	9044	1108	4867
All cities	2021	140	14876	1600	11886	1390	4938
All cities	2022	140	14254	3197	8677	2380	9586
All cities	2023	140	13537	3446	8205	1886	8864
All cities	2024	140	14019	3863	8154	2002	9558

Table S26A. Annual HLS source-inventory availability across all 140 fixed analytical units before pixel-level quality control. *Column panel 2 of 2; identifier columns are repeated.*

Geography	Year	S30 Acquisition Records	L30 Inclusive Share	S30 Inclusive Share	Classification Note
All cities	2013	0	1	0	Immutable source-inventory unit-days before pixel-level quality control; same-date L30 and S30 availability is classified as both.
All cities	2014	0	1	0	Immutable source-inventory unit-days before pixel-level quality control; same-date L30 and S30 availability is classified as both.
All cities	2015	0	1	0	Immutable source-inventory unit-days before pixel-level quality control; same-date L30 and S30 availability is classified as both.
All cities	2016	7924	0.394698	0.66707	Immutable source-inventory unit-days before pixel-level quality control; same-date L30 and S30 availability is classified as both.
All cities	2017	10263	0.318943	0.751771	Immutable source-inventory unit-days before pixel-level quality control; same-date L30 and S30 availability is classified as both.
All cities	2018	17766	0.241727	0.855857	Immutable source-inventory unit-days before pixel-level quality control; same-date L30 and S30 availability is classified as both.
All cities	2019	15372	0.262556	0.842927	Immutable source-inventory unit-days before pixel-level quality control; same-date L30 and S30 availability is classified as both.
All cities	2020	16078	0.24539	0.847059	Immutable source-inventory unit-days before pixel-level quality control; same-date L30 and S30 availability is classified as both.
All cities	2021	20346	0.200995	0.892444	Immutable source-inventory unit-days before pixel-level quality control; same-date L30 and S30 availability is classified as both.
All cities	2022	17863	0.391259	0.775712	Immutable source-inventory unit-days before pixel-level quality control; same-date L30 and S30 availability is classified as both.
All cities	2023	16076	0.393883	0.745438	Immutable source-inventory unit-days before pixel-level quality control; same-date L30 and S30 availability is classified as both.
All cities	2024	16008	0.418361	0.724445	Immutable source-inventory unit-days before pixel-level quality control; same-date L30 and S30 availability is classified as both.

Table S26B. Annual HLS source-inventory availability by analytical city before pixel-level quality control. *Column panel 1 of 2; identifier columns are repeated.*

Geography	Year	Unit Year Records	Inventory Unit Days	L30 Only Unit Days	S30 Only Unit Days	Both Sensor Unit Days	L30 Acquisition Records
Berlin	2013	60	1128	1128	0	0	2548
Berlin	2014	60	1212	1212	0	0	2747
Berlin	2015	60	1387	1387	0	0	3123
Berlin	2016	60	3384	1034	2110	240	2882
Berlin	2017	60	3705	933	2520	252	2692
Berlin	2018	60	5587	895	4143	549	3264
Berlin	2019	60	4727	850	3372	505	3044
Berlin	2020	60	5129	714	3900	515	2764
Berlin	2021	60	6880	598	5657	625	2766
Berlin	2022	60	6484	1508	3924	1052	5790
Berlin	2023	60	5696	1463	3438	795	5093
Berlin	2024	60	5759	1489	3480	790	5145
Madrid	2013	20	241	241	0	0	253
Madrid	2014	20	304	304	0	0	319
Madrid	2015	20	323	323	0	0	339
Madrid	2016	20	936	181	715	40	232
Madrid	2017	20	1368	221	1067	80	316
Madrid	2018	20	1915	142	1673	100	254
Madrid	2019	20	2004	180	1704	120	315
Madrid	2020	20	1922	201	1621	100	316
Madrid	2021	20	2006	220	1685	101	337
Madrid	2022	20	2206	385	1617	204	638
Madrid	2023	20	2174	388	1601	185	601
Madrid	2024	20	2246	365	1637	244	639
Vienna	2013	60	1304	1304	0	0	1655
Vienna	2014	60	1151	1151	0	0	1460
Vienna	2015	60	1353	1353	0	0	1722
Vienna	2016	60	3111	1259	1673	179	1814
Vienna	2017	60	3822	1054	2471	297	1711
Vienna	2018	60	5492	836	4037	619	1850
Vienna	2019	60	4996	812	3572	612	1805
Vienna	2020	60	4934	918	3523	493	1787
Vienna	2021	60	5990	782	4544	664	1835
Vienna	2022	60	5564	1304	3136	1124	3158
Vienna	2023	60	5667	1595	3166	906	3170
Vienna	2024	60	6014	2009	3037	968	3774
All cities	2013	140	2673	2673	0	0	4456
All cities	2014	140	2667	2667	0	0	4526
All cities	2015	140	3063	3063	0	0	5184
All cities	2016	140	7431	2474	4498	459	4928
All cities	2017	140	8895	2208	6058	629	4719

Geography	Year	Unit Year Records	Inventory Unit Days	L30 Only Unit Days	S30 Only Unit Days	Both Sensor Unit Days	L30 Acquisition Records
All cities	2018	140	12994	1873	9853	1268	5368
All cities	2019	140	11727	1842	8648	1237	5164
All cities	2020	140	11985	1833	9044	1108	4867
All cities	2021	140	14876	1600	11886	1390	4938
All cities	2022	140	14254	3197	8677	2380	9586
All cities	2023	140	13537	3446	8205	1886	8864
All cities	2024	140	14019	3863	8154	2002	9558

Table S26B. Annual HLS source-inventory availability by analytical city before pixel-level quality control. *Column panel 2 of 2; identifier columns are repeated.*

Geography	Year	S30 Acquisition Records	L30 Inclusive Share	S30 Inclusive Share	Classification Note
Berlin	2013	0	1	0	Immutable source-inventory unit-days before pixel-level quality control; same-date L30 and S30 availability is classified as both.
Berlin	2014	0	1	0	Immutable source-inventory unit-days before pixel-level quality control; same-date L30 and S30 availability is classified as both.
Berlin	2015	0	1	0	Immutable source-inventory unit-days before pixel-level quality control; same-date L30 and S30 availability is classified as both.
Berlin	2016	4836	0.376478	0.694444	Immutable source-inventory unit-days before pixel-level quality control; same-date L30 and S30 availability is classified as both.
Berlin	2017	5684	0.319838	0.748178	Immutable source-inventory unit-days before pixel-level quality control; same-date L30 and S30 availability is classified as both.
Berlin	2018	10131	0.258457	0.839807	Immutable source-inventory unit-days before pixel-level quality control; same-date L30 and S30 availability is classified as both.
Berlin	2019	8317	0.286651	0.820182	Immutable source-inventory unit-days before pixel-level quality control; same-date L30 and S30 availability is classified as both.
Berlin	2020	9314	0.239618	0.860792	Immutable source-inventory unit-days before pixel-level quality control; same-date L30 and S30 availability is classified as both.
Berlin	2021	12224	0.177762	0.913081	Immutable source-inventory unit-days before pixel-level quality control; same-date L30 and S30 availability is classified as both.
Berlin	2022	10652	0.394818	0.767428	Immutable source-inventory unit-days before pixel-level quality control; same-date L30 and S30 availability is classified as both.
Berlin	2023	9141	0.396419	0.743153	Immutable source-inventory unit-days before pixel-level quality control; same-date L30 and S30 availability is classified as both.
Berlin	2024	9024	0.395728	0.741448	Immutable source-inventory unit-days before pixel-level quality control; same-date L30 and S30 availability is classified as both.
Madrid	2013	0	1	0	Immutable source-inventory unit-days before pixel-level quality control; same-date L30 and S30 availability is classified as both.
Madrid	2014	0	1	0	Immutable source-inventory unit-days before pixel-level quality control; same-date L30 and S30 availability is classified as both.
Madrid	2015	0	1	0	Immutable source-inventory unit-days before pixel-level quality control; same-date L30 and S30 availability is classified as both.
Madrid	2016	788	0.236111	0.806624	Immutable source-inventory unit-days before pixel-level quality control; same-date L30 and S30 availability is classified as both.
Madrid	2017	1203	0.220029	0.83845	Immutable source-inventory unit-days before pixel-level quality control; same-date L30 and S30 availability is classified as both.
Madrid	2018	1859	0.126371	0.925849	Immutable source-inventory unit-days before pixel-level quality control; same-date L30 and S30 availability is classified as both.
Madrid	2019	1911	0.149701	0.91018	Immutable source-inventory unit-days before pixel-level quality control; same-date L30 and S30 availability is classified as both.
Madrid	2020	1804	0.156608	0.895421	Immutable source-inventory unit-days before pixel-level quality control; same-date L30 and S30 availability is classified as both.
Madrid	2021	1874	0.16002	0.890329	Immutable source-inventory unit-days before pixel-level quality control; same-date L30 and S30 availability is classified as both.
Madrid	2022	1911	0.266999	0.825476	Immutable source-inventory unit-days before pixel-level quality control; same-date L30 and S30 availability is classified as both.
Madrid	2023	1871	0.263569	0.821527	Immutable source-inventory unit-days before pixel-level quality control; same-date L30 and S30 availability is classified as both.
Madrid	2024	1971	0.271149	0.837489	Immutable source-inventory unit-days before pixel-level quality control; same-date L30 and S30 availability is classified as both.
Vienna	2013	0	1	0	Immutable source-inventory unit-days before pixel-level quality control; same-date L30 and S30 availability is classified as both.

Geography	Year	S30 Acquisition Records	L30 Inclusive Share	S30 Inclusive Share	Classification Note
Vienna	2014	0	1	0	Immutable source-inventory unit-days before pixel-level quality control; same-date L30 and S30 availability is classified as both.
Vienna	2015	0	1	0	Immutable source-inventory unit-days before pixel-level quality control; same-date L30 and S30 availability is classified as both.
Vienna	2016	2300	0.462231	0.595307	Immutable source-inventory unit-days before pixel-level quality control; same-date L30 and S30 availability is classified as both.
Vienna	2017	3376	0.35348	0.724228	Immutable source-inventory unit-days before pixel-level quality control; same-date L30 and S30 availability is classified as both.
Vienna	2018	5776	0.264931	0.847779	Immutable source-inventory unit-days before pixel-level quality control; same-date L30 and S30 availability is classified as both.
Vienna	2019	5144	0.285028	0.83747	Immutable source-inventory unit-days before pixel-level quality control; same-date L30 and S30 availability is classified as both.
Vienna	2020	4960	0.285975	0.813944	Immutable source-inventory unit-days before pixel-level quality control; same-date L30 and S30 availability is classified as both.
Vienna	2021	6248	0.241402	0.869449	Immutable source-inventory unit-days before pixel-level quality control; same-date L30 and S30 availability is classified as both.
Vienna	2022	5300	0.436377	0.765636	Immutable source-inventory unit-days before pixel-level quality control; same-date L30 and S30 availability is classified as both.
Vienna	2023	5064	0.441327	0.718546	Immutable source-inventory unit-days before pixel-level quality control; same-date L30 and S30 availability is classified as both.
Vienna	2024	5013	0.495012	0.665946	Immutable source-inventory unit-days before pixel-level quality control; same-date L30 and S30 availability is classified as both.
All cities	2013	0	1	0	Immutable source-inventory unit-days before pixel-level quality control; same-date L30 and S30 availability is classified as both.
All cities	2014	0	1	0	Immutable source-inventory unit-days before pixel-level quality control; same-date L30 and S30 availability is classified as both.
All cities	2015	0	1	0	Immutable source-inventory unit-days before pixel-level quality control; same-date L30 and S30 availability is classified as both.
All cities	2016	7924	0.394698	0.66707	Immutable source-inventory unit-days before pixel-level quality control; same-date L30 and S30 availability is classified as both.
All cities	2017	10263	0.318943	0.751771	Immutable source-inventory unit-days before pixel-level quality control; same-date L30 and S30 availability is classified as both.
All cities	2018	17766	0.241727	0.855857	Immutable source-inventory unit-days before pixel-level quality control; same-date L30 and S30 availability is classified as both.
All cities	2019	15372	0.262556	0.842927	Immutable source-inventory unit-days before pixel-level quality control; same-date L30 and S30 availability is classified as both.
All cities	2020	16078	0.24539	0.847059	Immutable source-inventory unit-days before pixel-level quality control; same-date L30 and S30 availability is classified as both.
All cities	2021	20346	0.200995	0.892444	Immutable source-inventory unit-days before pixel-level quality control; same-date L30 and S30 availability is classified as both.
All cities	2022	17863	0.391259	0.775712	Immutable source-inventory unit-days before pixel-level quality control; same-date L30 and S30 availability is classified as both.
All cities	2023	16076	0.393883	0.745438	Immutable source-inventory unit-days before pixel-level quality control; same-date L30 and S30 availability is classified as both.
All cities	2024	16008	0.418361	0.724445	Immutable source-inventory unit-days before pixel-level quality control; same-date L30 and S30 availability is classified as both.

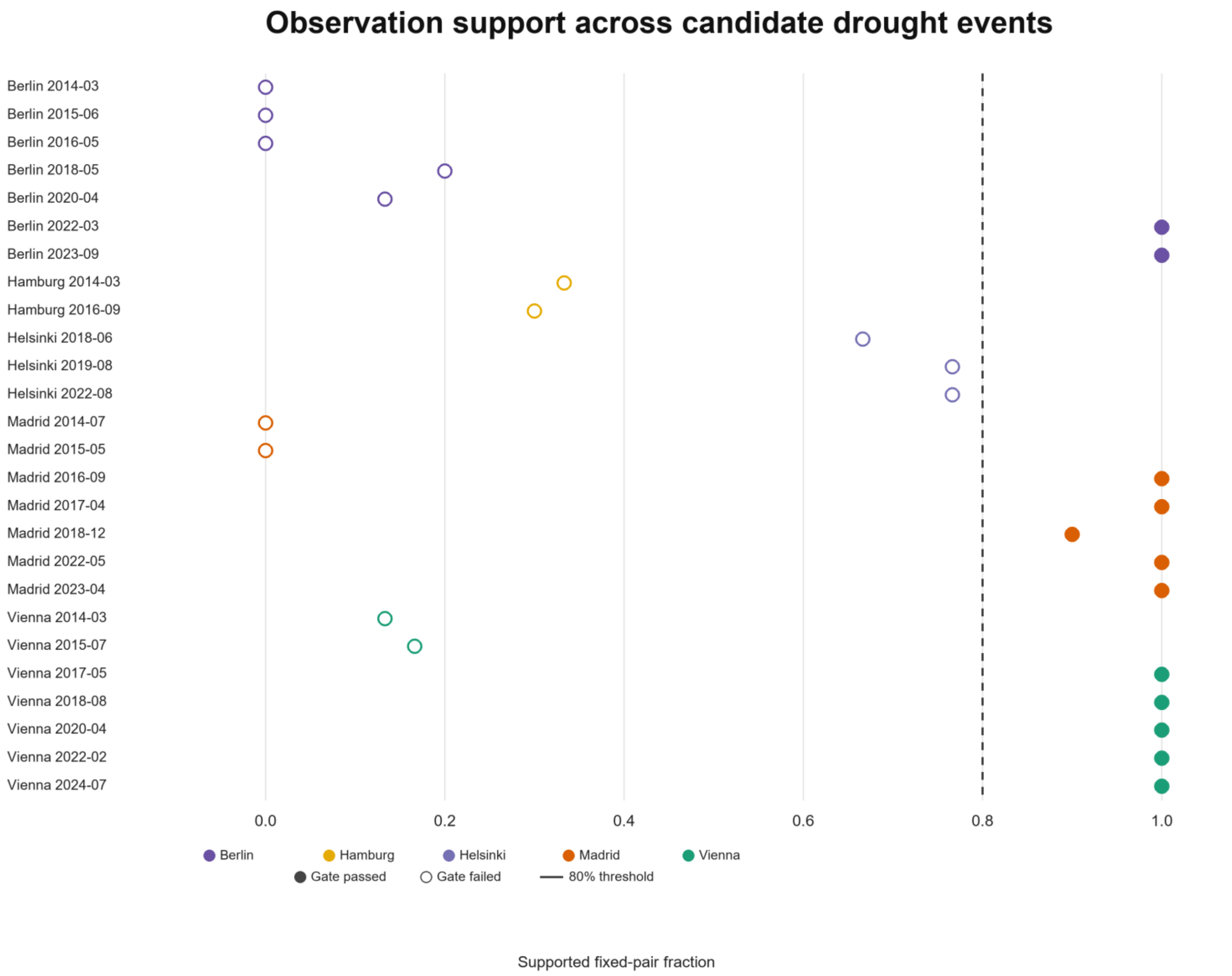

Figure S1. Supported-pair distributions. Supported fixed-pair fractions are shown for all 26 candidate events. The dashed line marks the unchanged 80% event-level observation-support threshold. Filled points passed the gate, open points did not, and colours distinguish cities.

Within-event pair dispersion in the primary equal-month outcome

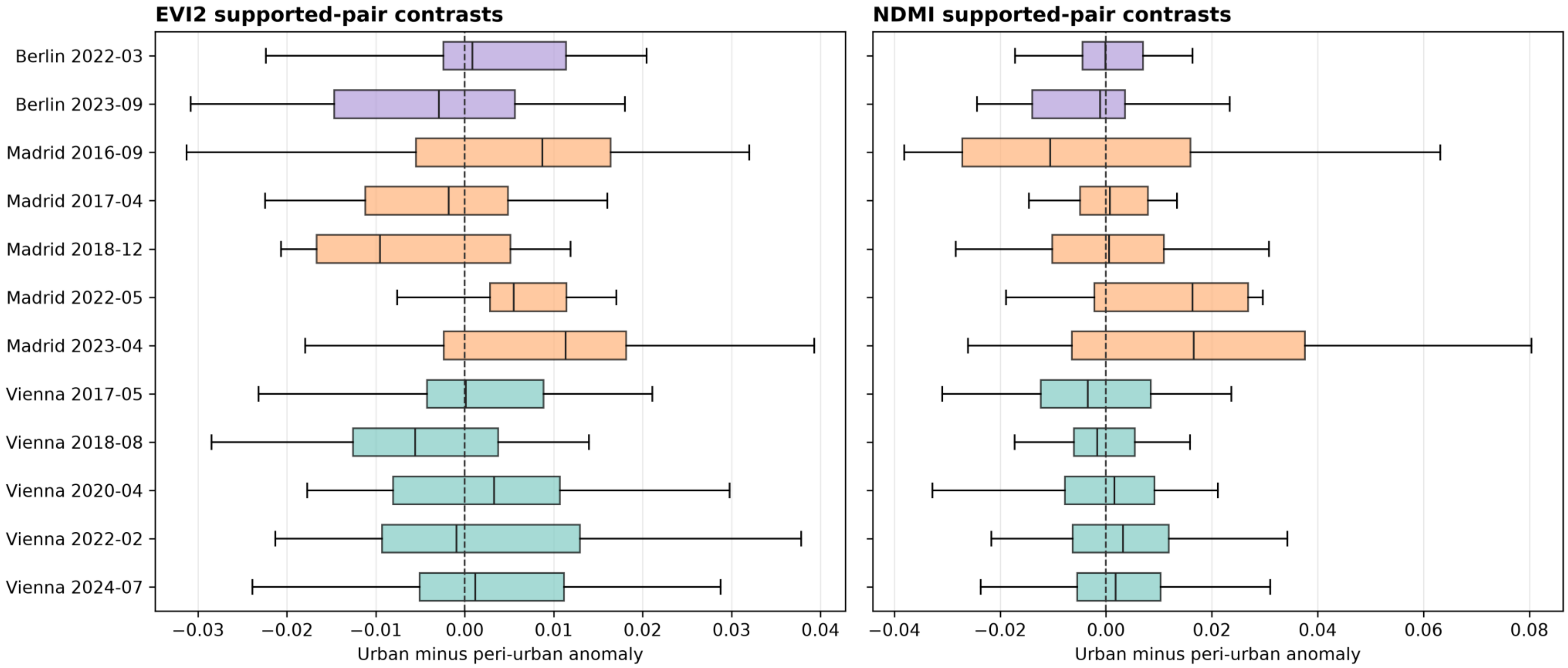

Figure S2. Pair-level outcome distributions. Boxplots show supported-pair urban-minus-peri-urban contrasts for EVI2 and NDMI within each retained event. Boxes span the interquartile range, centre lines mark medians, whiskers show the non-outlier range, the dashed line marks zero, and colours distinguish cities.

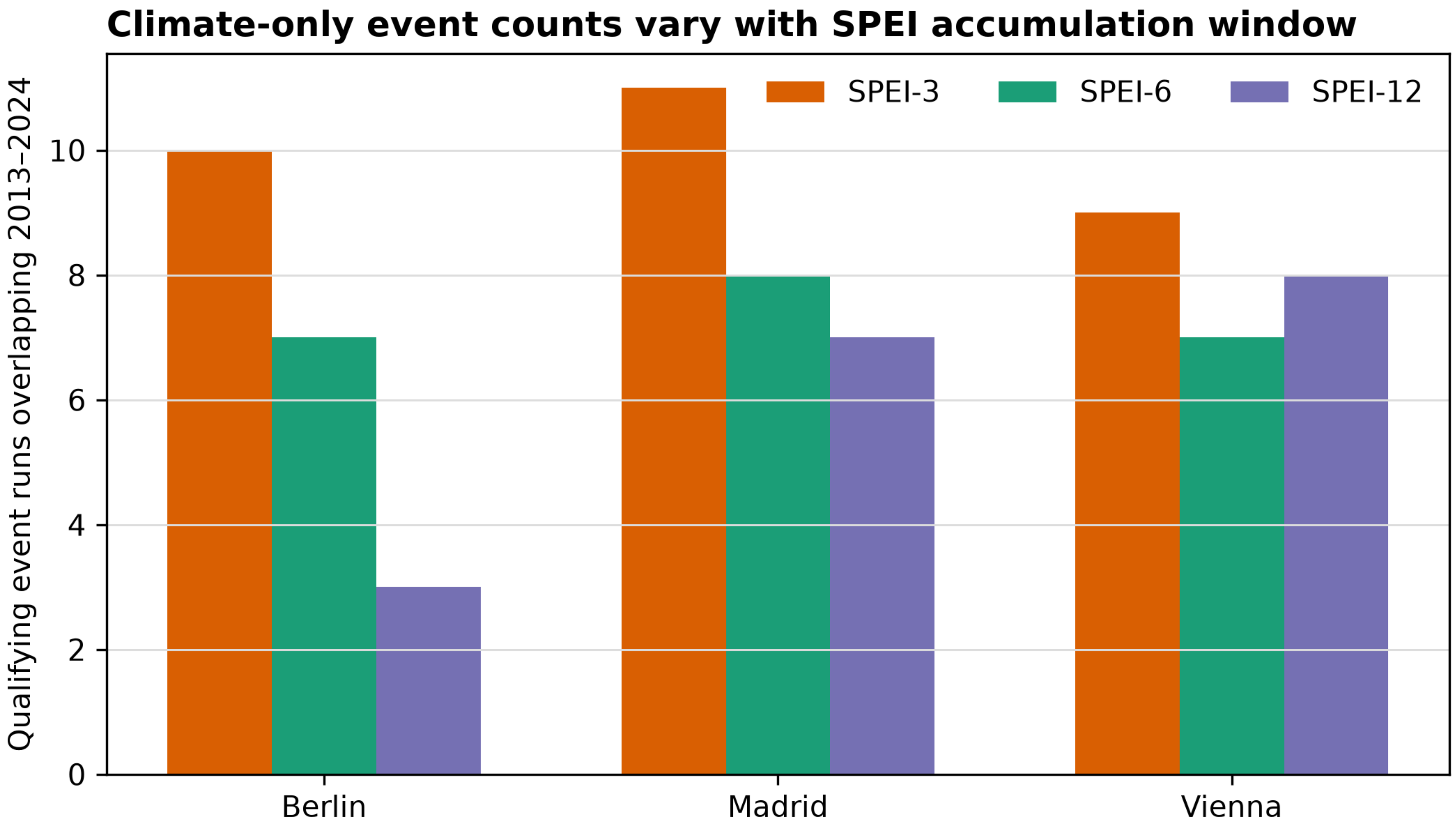

Figure S3. Climate-only SPEI-scale event inventory. Bars show qualifying drought runs overlapping the 2013-2024 HLS response record for SPEI accumulation windows of 3, 6, and 12 months. Vegetation outcomes were not re-estimated for alternative accumulation windows.
